# Recent Advances in the Interfacial Shear and Dilational Rheology of Polymer Systems: From Fundamentals to Applications

**DOI:** 10.3390/polym14142844

**Published:** 2022-07-13

**Authors:** Younes El Omari, Mohamed Yousfi, Jannick Duchet-Rumeau, Abderrahim Maazouz

**Affiliations:** 1 Université de Lyon, CNRS, UMR 5223, Ingénierie des Matériaux Polymères, Université Claude Bernard Lyon 1, INSA Lyon, Université Jean Monnet, CEDEX, F-69621 Villeurbanne, France; younes.el-omari@insa-lyon.fr (Y.E.O.); jannick.rumeau@insa-lyon.fr (J.D.-R.); abderrahim.maazouz@insa-lyon.fr (A.M.); 2 Hassan II Academy of Science and Technology, Rabat 69621, Morocco

**Keywords:** interfacial shear rheology, interfacial dilational rheology, double-wall ring, bicone, oscillating drop tensiometry

## Abstract

The study of the viscoelastic properties of polymer systems containing huge internal two-dimensional interfacial areas, such as blends, foams and multilayer films, is of growing interest and plays a significant role in a variety of industrial fields. Hence, interfacial rheology can represent a powerful tool to directly investigate these complex polymer–polymer interfaces. First, the current review summarizes the theoretical basics and fundamentals of interfacial shear rheology. Particular attention has been devoted to the double-wall ring (DWR), bicone, Du Noüy ring and oscillating needle (ISR) systems. The measurement of surface and interfacial rheological properties requires a consideration of the relative contributions of the surface stress arising from the bulk sub-phases. Here, the experimental procedures and methodologies used to correct the numerical data are described considering the viscoelastic nature of the interface. Second, the interfacial dilational rheology is discussed, starting with the theory and underlying principles. In particular, the Langmuir trough method, the oscillating spinning drop technique and the oscillating pendant drop technique are investigated. The major pioneering studies and latest innovations dedicated to interfacial rheology in both shear and dilatation–compression are highlighted. Finally, the major challenges and limits related to the development of high-temperature interfacial rheology at the molten state are presented. The latter shows great potential for assessing the interfaces of polymer systems encountered in many high-value applications.

## 1. Introduction

Polymers are rarely miscible because of their high viscosity. When two immiscible polymers meet, a surface called an “interface” is defined. This surface is thermodynamically created due to the positive free enthalpy of mixing (Gibbs) related to the structural properties of the polymers. In particular, it is due to the low entropy of the macromolecular chains (low number of possible mixed configurations) and the poor chemical affinity (repulsive interactions) between the polymer constituents. The “interface” can be described as an “interphase,” which is a tridimensional entity. We make must a distinction between a physical interphase and a chemical interphase. A physical interphase is created when the two coexisting polymers are fully or partially miscible, or when prepared by forced assembly in the molten state, resulting in a physical intra-entanglement as in the coextrusion process [1]. A chemical interphase is created when a chemical reaction occurs at the interface of these two polymers, forming an interfacial copolymer [2].

The interfacial properties in multiphase polymer systems can control their morphology (e.g., a dispersion of one polymer phase within another polymer phase, coalescence) [3], their stability (e.g., emulsions [4], foams [5], polymer solutions [6]), their reinforcement (e.g., polymer/clay nanocomposites [7]) and their healing degree (e.g., multi-layered polymers [8]). Interfacial rheology is, thus, a powerful way to probe the polymer systems in different fields, including crude oil recovery [9], plastic processing [10], cosmetics [11], biomedical applications [12] and foods [13].

Interfacial rheology directly investigates the viscoelastic responses of the interface under the application of a stress or strain (i.e., the same formalism and techniques developed in bulk rheology). This two-dimensional area possesses properties that differ from those of the two sub-phases. This interface can be considered the third phase and is characterized not only by its interfacial tension but also by its rheological properties. The latter can affect the overall system [14].

The extensive state-of-the-art research reveals that most experimental studies devoted to interfacial rheology have concerned high flow rate liquid systems (suspensions, emulsions, etc.). However, reports investigating the particular case of high molecular weight polymer systems are not plentiful. This is related to the constraints that occur during the study of polymers, such as the viscosity, high inertia or fragility of the interfacial fixtures and the temperature limit (measurements are often made below 80 °C to ensure a stable, homogeneous temperature at the interface). Developing new interfacial rheological setups is a subject of increasing interest in the industrial and academic communities.

Hence, a large section of the present paper is dedicated to these issues. First, the sufficient theoretical models and concepts related to the interfacial rheology during shear and dilatation or compression have been reviewed.

A good deal of the present paper details the methods of correction of the apparent measured interfacial properties in both oscillatory and steady-flow modes, taking into account the contributions of the sub-phases during the gathering of the numerical data.

Finally, this review ends by summarizing the major pioneering studies, experimental techniques and related applications of the interfacial rheology of polymer systems.

## 2. History

The term “rheology”, invented by Bingham in 1928, refers to the study of material movement and deformation in both fluid and solid states. In other words, it is the analysis of deformations under the impact of applied stress. In particular, rheologists have studied the rheology of different kinds of fluids, such as molten polymers, to better understand their processability and to monitor their industrial-scale implementation.

The study of multiphase systems is of great importance in academia, as well as in industry. From this perspective, it has been extremely valuable to study the deformation of their interfaces through interfacial rheology, i.e., the study of the stress–strain relationships taking place within interfaces, in the two-dimensional area located just between the two immiscible fluids, such as the interfacial layer of the surfactant or in the solid particles.

The term “interfacial viscosity” was introduced by Plateau in 1869 [15] to explain the slowing of the oscillation of a needle on the surface of water (gas–water surface). In the beginning, Plateau thought that he had used pure water, but this was not the case. The origin of these findings was related to the presence of impurities that remained on the surface of the water. He also described the surface gradient mechanisms that can be at the origin of convection movement, now known as the “Marangoni effect” [16].

**Figure 1:**
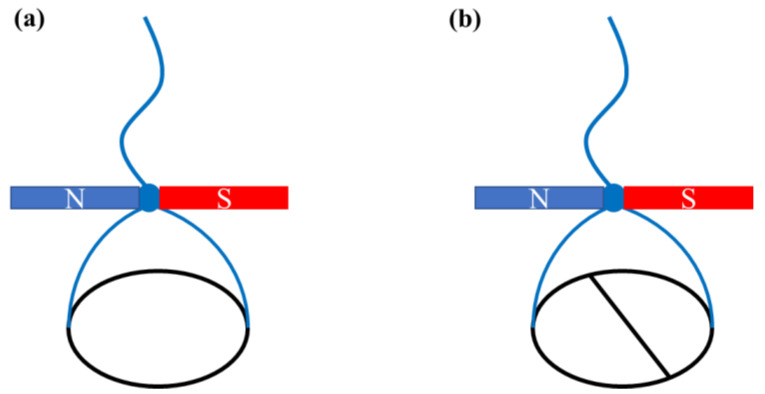
(**a**) Rayleigh’s experiment involving a ring of thin brass wire placed on the surface of the water and dusted over with fine sulfur. The system was magnetically rotated using a magnetized sewing needle (NS). (**b**) A material diameter of the same brass wire was added to the ring. Reprinted with permission from Ref. [17]. Copyright 1890 Copyright Royal Society.

Next, Rayleigh [17] confirmed Plateau’s assumption. He positioned a ring of thin brass wire on the surface of water dusted over with fine sulfur and set the system into rotation using an external magnet, namely a magnetized sewing needle (Figure 1a). He found no noticeable movement on the surface around the ring, and argued that an ordinary water surface does not appreciably resist the shear. Then, he slightly modified the apparatus by adding a material diameter of the same brass wire to the ring (Figure 1b), and found that the sulfur indicated that the entire water surface included within the semicircles now shared in the motion. In this case, Marangoni’s assumptions were also shown to be correct.

**Figure 2:**
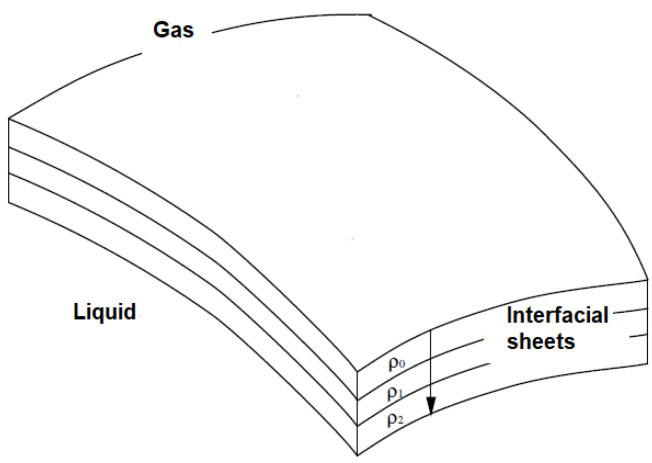
The Newtonian interface according to the Boussinesq model: pressure equalization by the density gradient.

In 1913, Boussinesq [18] used the term “surface viscosity” to explain the measured drop in velocity of a drop of mercury in castor oil, which differs from that predicted by the theory when the dissipation of the interface is neglected. Many models have described the interface between two immiscible liquids, such as those proposed by Boussinesq [18], Kasperski [19] and Shikhmurzaev [20]. Here, we present the Boussinesq model. In the static equilibrium, the isotropy in the volume is maintained by molecular agitation and results in the presence of a pressure field. Boussinesq imagined the surface as a superposition of layers with variable density (**ρ_0_**, **ρ_1_**, **ρ_2_**) (Figure 2). This density gradient gives enough force to the fluid to maintain it within a fixed range [21].

## 3. Vectors and Tensors on Interfaces

The flow in the liquid phases that establish the interface is described by conservation equations for the mass (continuity equation, Equation (1)), momentum (Navier–Stokes equation, Equation (2)) and energy.

(1) $$\frac{\partial \rho }{\partial t}+\nabla .(\rho v)=0$$

(2) $$\rho (\frac{\partial v}{\partial t}+v.\nabla v)=-\;\nabla p+\eta_{b}\nabla^{2}v+f\;$$

where **ρ** is the density, **t** is the time, **v** is the velocity, **p** is the pressure, $\eta_{b}$ is the bulk viscosity and **f** is the external body force.

**Figure 3:**
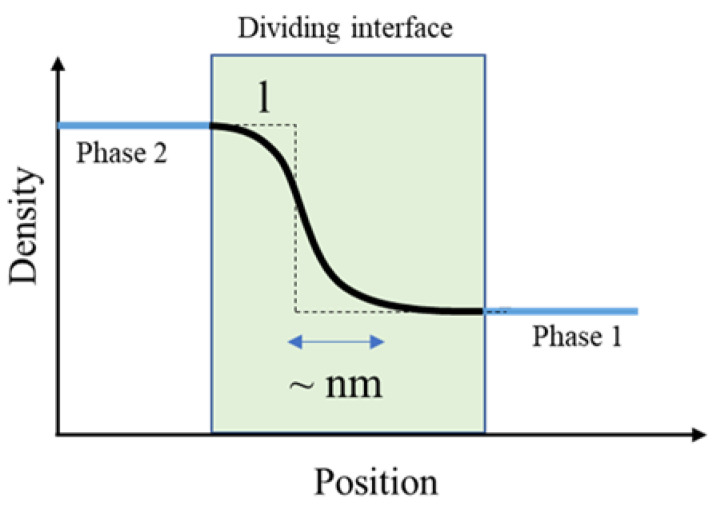
Schematic plot of the density **ρ** as a function of position ***x*** crossing the interface.

The question that one might ask now is the position of the interface. The location of the dividing surface is not unique. Gibbs described an interface as a two-dimensional dividing area embedded in a 3D space that is located between two bulk liquids. We can refer to this as a sharp interface (few nanometer~nm) [22]. Figure 3 shows that the bulk properties are extrapolated up to this surface, and any excess quantity that is not accounted for by the bulk is assigned to the interface. The most transparent approach is to set the location of the interface by considering **ρ_s_** = 0.

The properties of the interface are important parameters to define. According to the studies by Boussinesq, in addition to the surface tension **γ**, the interfacial stress **τ_s_** (Equation (3)) is a function of another intrinsic property of the interface, **τ_e_**, which is the extra stress corresponding to the interfacial rheological properties.

(3) $$\tau_{s}=\gamma .I_{s}+\tau_{e}\;$$

**Figure 4:**
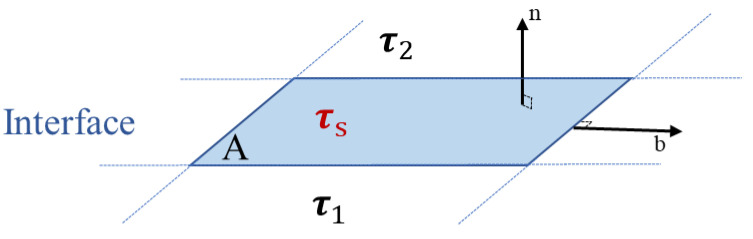
The surface element **A** separated two fluids denoted 1 and 2. The unit vectors normal and tangential to the surface are denoted by **n** and **b**, respectively.

In addition to the kinematic condition, the free surface must satisfy the conditions of continuity of the velocity and stress across the interface. The excess momentum balance equation is obtained by collecting all of the forces that act on interfacial element A (Figure 4) and identifying them as sources of momentum, starting with the interfacial inertia (left side of Equation (4)), the bulk stress (**τ_1_** and **τ_2_**) and the interfacial stress (right side of Equation (4)) [23].

(4) $$\iint_{A}\rho_{s}\frac{D_{s}v}{Dt}\;dA=\iint_{A}(\tau_{2}-\tau_{1}).ndA+\iint_{A}\nabla_{s}.\tau_{s}dA$$

where $\frac{D_{s}()}{\mathrm{Dt}}$ is the time derivative.

The interfacial inertia is generally neglected in the case of a sharp interface (**ρ_s_** = 0). By substituting the expression for the interfacial stress tensor into Equation (4), we obtain the following equation:

(5) $$(\tau_{1}-\tau_{2}).n=\nabla_{s}\gamma -\gamma \left(\nabla_{s}.n\right)n+\nabla_{s}.\tau_{e}=0$$

representing the bulk stress, Marangoni stress, and extra-capillarity surface stress.

Equation (5) links the transport phenomena (equation of state, i.e., **γ**(Г(t,x,y,z)) with the interfacial and bulk rheology (constitutive equations).

In 1959, Scriven [24] proposed a constitutive model describing the response of a purely Newtonian interface based on the Newton–Cauchy–Poisson law. This interface has thermodynamic properties corresponding to the surface (or interfacial) tension and rheological properties based on the interfacial dilatational viscosity (Pa.s.m) and the interfacial shear viscosity (Pa.s.m). This constitutive equation is known as the generalized Boussinesq–Scriven surface fluid model. This model describes the surface (or interfacial) stress tensor **τ_s_** (with units of force per unit length) as a linear function of the surface rate of the deformation tensor $\bar{D_{s}}\;$and can be expressed as:

(6) $$\tau_{e}\;=[(\eta_{d}-\eta_{s})\;{\mathrm{div}}_{s}{\bar{V}}_{s}].I_{s}+2\eta_{s}\;\bar{D_{s}}\;$$

(7) $$\bar{D_{s}}\;=\frac{\nabla_{s}{\bar{V}}_{s}.I_{s}+I_{s}\;.\;{\left(\nabla_{s}{\bar{V}}_{s}\right)}^{T}\;}{2}\;$$

This constitutive equation includes ***η*_d_**, the surface dilatational viscosity; ***η*_s_**, the surface shear viscosity; **γ**, the thermodynamic (equilibrium) surface (or interfacial) tension;${\;I}_{s}$, the surface unit tensor; **τ_e_**, the extra stress component due to the deformation tensor;$\;\bar{V}$**_s_**, the surface (or interfacial) velocity vector; **div_s_**, the surface (or interfacial) divergence operator; $\nabla_{s}$, the surface (or interfacial) gradient operator; and $\bar{D_{s}}$**,** the tensor of the surface deformation rate. Note that $\bar{V}$**_s_** has normal and tangential components to the interface. The Boussinesq–Scriven model describes a Newtonian interface. It has limited functionality for non-Newtonian interfaces, because they exhibit a complex, non-linear stress–strain relationship.

The behavior of a solid-like elastic surface can be described by a linear elastic model [24,25] identical to Equation (6), which is valid for infinitely small deformations.

(8) $$\tau_{e}=[(K_{s}-G_{s})\;{\mathrm{div}}_{s}\;\bar{V}s].I_{s}+2G_{s}\bar{U_{s}}\;$$

(9) $$\;\bar{U_{s}}\;=\frac{\nabla_{s}{\bar{V}}_{s}.I_{s}+I_{s}\;.{\left(\nabla_{s}{\bar{V}}_{s}\right)}^{T}\;}{2}\;$$

where **K_s_** is the interfacial dilatational modulus, **G_s_** is the interfacial shear modulus, **v_s_** is the displacement vector on the interface and **U_s_** is the interfacial infinitesimal strain tensor. Note that these two constitutive equations describe the behavior of purely viscous and purely elastic plane interfaces. Another tensor projection P onto the interface needs to be performed for the general case of a curved interface with regular components in the interfacial velocity or displacement [26]. Pepicelli et al. [27] recently developed a “neo-Hookean” constitutive equation from a strain energy function, which separates the dilatational or compressional contributions from the contributions related to shear deformations, yielding the interfacial or surface elastic stress tensor. With all of these equations, as in 3D rheology, developing a modeling equation as well as numerical methods for a complex interface becomes possible and remains a task in progress [23,26,28,29,30,31,32,33].

From an experimental point of view, a sinusoidal deformation is often applied in order to characterize viscoelastic interfaces, because in rheology, time becomes an essential variable in the experiment. The principle and interpretation of the interfacial analysis depend on the type of stress applied (shear or dilatation) and on the nature of the deformation (constant speed deformation or sinusoidal deformation). In the current review, particular attention has been dedicated to summarizing the theoretical basics and principles of the devices used to study interfacial shear and dilational rheology.

## 4. Interfacial Shear Rheology

This analysis consists of deforming the interface (without changing its area) by moving an object with variable geometry (needle, bi-cone, ring, etc.). The resistance of the interface is recorded by the sensor of the rheometer. The solicitation mode (force or torque) makes it possible to determine the interfacial stress **τ_s_** (N.m^−1^ or Pa.m). However, the object movement analysis makes it possible to determine the interfacial deformation ε_s_ (dimensionless magnitude) and the rate of the interfacial deformation $\dot{\varepsilon }$_s_ (s^−1^), which gives a rheogram **τ_s_** = f($\dot{\varepsilon }$_s_).

### 4.1. Interfacial Rheological Magnitudes

#### 4.1.1. With Imposed Stress (or Creep Measurement)

In static measurements, a constant stress **τ_0_** is applied, and the compliance **J (t)** (in m/N) is measured as:

(10) $$J\;(t)=\frac{\varepsilon \left(t\right)\;}{\tau_{0}}$$

where **ε(t)** is the interfacial deformation.

In harmonic measurements, a sinusoidal stress with magnitude **τ_0_** is applied, and the complex compliance J*(ω) is measured with:

J*(ω) = J′-i J″ (11)

#### 4.1.2. With Imposed Strain (or Relaxation Measurement)

In static measurements, a constant strain **ε_0_** is applied, and the interfacial shear modulus **G (t)** (in N m) is measured:

(12) $$G\;(t)=\frac{\tau \left(t\right)\;}{\varepsilon_{0}}$$

where **τ(t)** is the interfacial stress.

In harmonic measurements, on the other hand, a sinusoidal strain with magnitude **ε_0_** is applied, and the complex interfacial shear modulus G*(ω) is measured:

(13) $$G*(\omega )=G^{\prime }\;(\omega )+{\mathrm{iG}}^{\prime \prime }\;(\omega )=\;\left|G^{*}\right|e^{i\delta \left(\omega \right)}=-i\omega \eta *(\omega )$$

The G* modulus is expressed via its real and imaginary components, which depend on the oscillation frequency. In this case, the dimension of the components of the complex modulus is also N/m or Pa.m. The real component **G′**, which is in phase with the applied deformation profiles, characterizes the elastic properties, while the imaginary component **G″**, which is out of phase with the applied deformation profiles, describes the viscous properties of the interface.

Viscoelastic interfaces can be also described by the complex viscosity η*(Pa.s.m) and phase angle **δ** (°), where:

η*(**ω**)= ***η***′ (**ω**) − **i*η***″ (**ω**) (14)

And:

(15) $$\tan\;\delta =\;\frac{G^{\prime \prime }\left(\omega \right)}{G^{\prime }\left(\omega \right)}\;$$

### 4.2. Positioning of the Shear Geometry at the Interface

**Figure 5:**
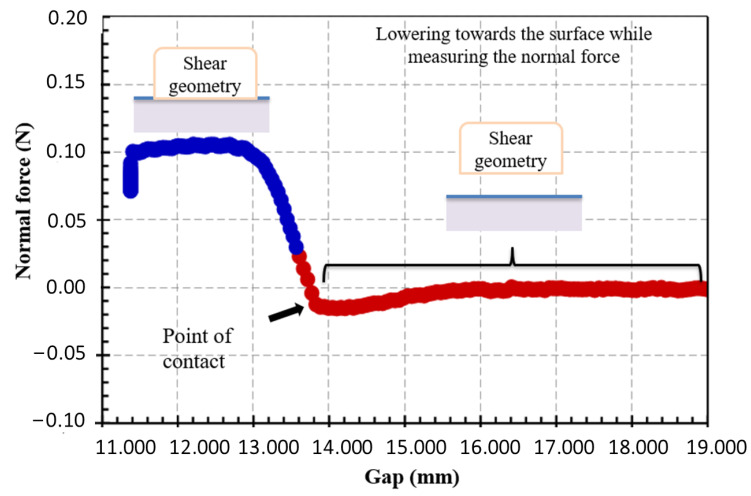
Positioning of the object at the interface with normal force assistance.

Presently, there are two methods for positioning the shear geometry at the interface. In the first method, the geometry can be positioned at the interface using a technique called normal force assistance (Figure 5). The geometry is lowered from a known gap toward the surface while the rheometer measures the axial force. The normal force increases dramatically upon contact with the sub-phase surface of greater density.

**Figure 6:**
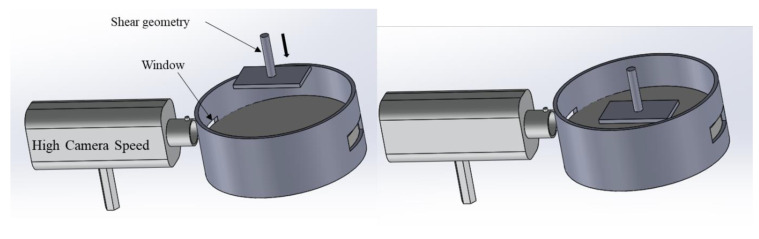
Positioning of the object at the interface using a visualization system. Reprinted from Ref. [35]. Copyright 2022 Copyright Clearance Center.

In the second method, the geometry can be positioned at the interface by means of a visualization system (Figure 6) [34] that is used to ensure that the edge of the geometry is precisely located at the interface.

In addition to the difficulty of positioning the shear geometry at the interface, making accurate measurements is not straightforward. This is related to the sensitivity of the instrument employed, i.e., the contact surface between the geometry and the interface. In general, the interfaces are very thin, making the measurement difficult due to the low interfacial measured torque T (a few mN.m), which is usually very close to the limits of the rheometer and is affected by the inertia of the instrument and of the geometry [36].

### 4.3. The Boussinesq Number

In surface rheology measurements, it is very important to realize that the rheometer is going to measure the total response (stress or strain), including the responses of the interface and the sub-phases. The measurement of the interfacial rheological properties makes it necessary to consider the relative contributions of the interfacial stress and the stress resulting from the bulk liquid. The ratio of these two stresses is described by the Boussinesq number (**Bo**) [37].

In other words, the **Bo** allows us to foresee if the effects of the sub-phases can be neglected with respect to the interfacial effect. The Boussinesq number can be expressed as follows (Equation (16)):

(16) $$\mathrm{Bo}\;=\;\;\frac{\mathrm{surface}\;\mathrm{drag}\;\mathrm{force}}{\mathrm{sub}-\mathrm{phases}\;\mathrm{drag}\;\mathrm{forces}}=\;\frac{\eta_{s}.P.\frac{V}{L_{s}}}{\eta_{v}.A.\frac{V}{L_{v}}}=\frac{\eta_{s}}{l.\eta_{v}}\;$$

where ***η*_s_** (Pa.s.m) is the surface viscosity in a steady shear flow that is calculated from the geometry adopted in the interfacial rheological measurements and from the measured torque; ***η*_v_** is the bulk viscosity (Pa.s) = (***η*_1_** + ***η*_2_**), with ***η*_1_** and ***η*_2_** representing the viscosities of sub-phases 1 and 2, respectively; **V** is the characteristic velocity (m/s); **L_s_** and **L_v_** are the characteristic length scales over which the characteristic velocity decays at the interface and in the sub-phases, respectively (m); **P** is the contact perimeter between the shear geometry and the interface (m); **A** is the contact area between the geometry and the surrounding sub-phases (m^2^).

The Boussinesq number is dimensionless. It is a product of two parts: the first one is a material part that is the ratio between the interfacial and bulk viscosities. The more viscous the sub-phases are, the higher their drag forces become. The second part is a geometrical one that depends on the ratio 1/1, which has units of length and defines a characteristic length scale ‘‘l’’ associated with the measurement techniques.

In practice, as ‘‘l’’ is mainly determined by the ratio of **A** to **P**, the Boussinesq number is simplified to:

(17) $$\mathrm{Bo}=\frac{\eta_{s}\cdot \;P}{\eta_{v}\cdot \;A}\;$$

The interface and sub-phases can independently show non-Newtonian behavior. The sub-phases or the interface can have a shear-rate-dependent viscosity, such as in a generalized Newtonian fluid or a non-linear viscoelastic fluid. In the case of oscillatory measurements, the viscosities will depend on the frequency and the Boussinesq number will turn into a complex number Bo* [38,39].

(18) $$\mathrm{Bo}*=\frac{\eta_{s}^{*}}{l.\eta_{v}^{*}}=\frac{G_{s}^{*}}{-l.\omega .i.\eta_{v}^{*}}=\frac{G^{\prime \prime }\left(\omega \right)-iG^{\prime }\left(\omega \right)}{l.\omega .\eta_{v}^{*}}$$

According to the value of **Bo**, there are two cases, as below.

If the **Bo** value is high (**Bo** >> 1), the contribution of the sub-phases to the interfacial flow is very negligible (i.e., very little fluid viscosity). This is true when the interface is rigid (high viscosity ***η*_s_**) or when the geometric ratio (1/1) is high (low contact area between the geometry and the interface).

If the **Bo** value is low (**Bo** << 1), the interfacial rheological properties could be overestimated due to the high contributions of the flow in the sub-phase. In such instances, specific numerical corrections need to be carried out to the measurable magnitudes [36,40].

### 4.4. Correction for the Contributions of the Sub-Phases

**Figure 7:**
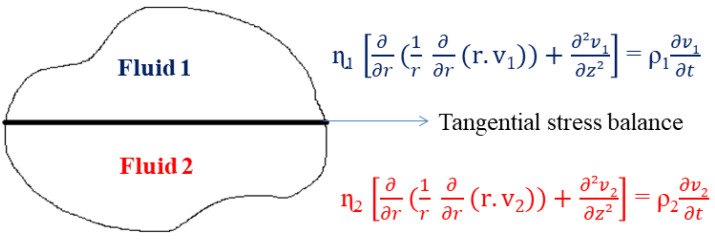
Tangential stress balance.

The velocity profiles v in the sub-phases and at the interface need to be calculated for different Boussinesq numbers in the cylindrical coordinate system (r, θ, z) in order to solve the Navier–Stokes equation (Figure 7) [36]. Supposing that the inertial effects are neglected; the interfaces are planar, incompressible and of very low Reynolds numbers; and the stress–deformation behavior of the liquid interface may be represented by the generalized Boussinesq surface fluid model (described above), then the velocity profiles can be obtained from the Navier–Stokes equation (based on the fundamental principle of dynamics (FDP)).

Approximations and boundary conditions:

- Boundary conditions:

1. No sliding at the walls of the tank and maximum speed at the walls of the geometry:

**v_s_ (Cup boundary) = 0 and v_s_ (Geometry boundary) = max**

2. Zero speed along z: v (z) = 0;
3. Boussinesq–Scriven model (balance of tangential stress) at the interface.

- The tangential stress balance condition is as follows (under steady-state conditions):

(19) $$\eta_{1}\;\frac{\partial v_{1}}{\partial z}\;-\;\eta_{2}\;\frac{\partial v_{2}}{\partial z}\;=\pm \eta_{s}\;\frac{\partial }{\partial r}\;(\frac{1}{r}\;\frac{\partial }{\partial r}(r.\mathrm{vs}))$$

The ± sign refers to the condition for the inner and outer gaps, respectively.

The surface viscosity ***η*_s_** should be replaced by the complex number *η*_s_* for oscillatory flow.

- Separation of variables:
  **v_j_ = a_j_(r,z).Ω** (20)
  **Ω = θ_0_.i.ω.e^iωt^** (21)

where **v_j_** is the velocity in phase **j**; **a_j_** is the amplitude of the velocity, which is a function of **r** and **z**; **Ω** is the angular velocity; **ω** is the frequency; and **θ_0_** is the angular velocity amplitude.

An iterative system that is based on the calculation of the velocity profiles is used for the subtraction of the contribution of the sub-phases. In the first step of the iteration, the authors calculate the initial value of the interfacial viscosity, based particularly on the formulas of equivalent geometry known as the double-wall ring [36] or the biconical bob geometry [40].

This often happens by evaluating a test inside a loop and imposing a convergence condition. The following steps share a common structure for the loop, starting with the first measured value of interfacial viscosity, called the “seed”:

- Enter the “seed” interfacial viscosity;
- Compute the drag force and solve the Navier–Stokes equation, respecting the previously mentioned approximations and boundary conditions;
- Extract the new value of the interfacial viscosity;
- Check the convergence condition;
- If the convergence condition is verified, end the loop; if not, go back to the first step by using the first corrected value of the interfacial viscosity as a seed value.

All of these steps allow the computation of the corrected value of the torque T_C_ and the interfacial viscosity for a steady flow using this equation:

(22) $$\eta_{s}^{k+1}=\eta_{s}^{k}.\frac{T_{A}}{T_{C}}$$

where T_A_ refers to the measured torque and k to the iteration step. The iteration is repeated until the deviation of the interfacial viscosity between two successive iterations is lower than 0.05% [40].

It is useful to mention that at high frequencies, the effect of fluid inertia (Reynolds number, Re) also needs to be taken into account in the corrections according to the methodology developed by Reynaert et al. [38].

Nowadays, in the rheometer market, many geometries allow the characterization of the interfacial rheological properties; these geometries are divided into two categories with two different measurement principles, namely translation and rotation. We are particularly interested here in three commercially available geometries: the oscillating needle, the bicone and the ring.

### 4.5. Interfacial Translational Rheometers

#### 4.5.1. Oscillating Needle

**Figure 8:**
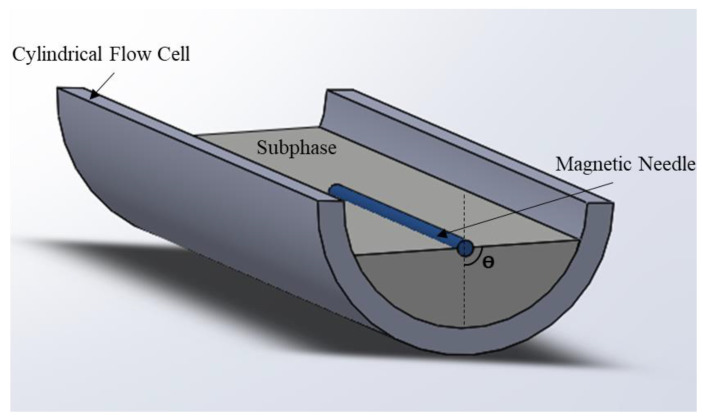
Schematic diagram of the oscillating needle device used for interfacial rheology.

The use of the oscillating needle as an interfacial rheological fixture was first proposed by Shahin [41]. The operating principle of the interfacial translational rheometer is practically the same as that of the bulk rheology. A magnetized needle is set up on the interface and oscillates longitudinally (Figure 8). This needle is aligned along with the flow cell (silanized hydrophobic glass capillary). The sheared interface is the horizontal surface between the needle and the edge of the capillary [41]. A gradient in the magnetic field is generated by applying a different current through the two Helmholtz coils, which drives (translates or oscillates) the magnetic needle along the glass channel, with the resulting needle position being captured by a camera [42].

**Figure 9:**
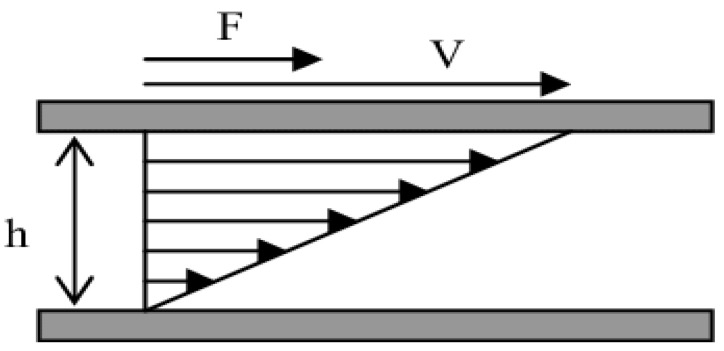
Shear between two parallel flat plates: one is fixed and the other is mobile.

The first oscillating needle was manufactured by Plateau [15]. Currently, it is marketed under the name of the Interfacial Shear Rheometer (ISR) (Figure 9). It can operate in two different modes: the static or harmonic regime.

In this case, the Boussinesq number can be expressed as:

(23) $$\mathrm{Bo}=\frac{P\eta_{s}}{A\;\eta_{v}}=\frac{\left(2L+4a\right)\eta_{s}}{2\left(\pi -\theta \right)\mathrm{aL}\eta_{v}}\;=\frac{\eta_{s}}{a\left(\pi -\theta \right)\eta_{v}}$$

where ***η*_s_** is the surface viscosity, ***η*_v_** is the viscosity of the bulk fluids, P is the perimeter of the probe in contact with the interface, A is the area of the probe in contact with the sub-phase, θ is the contact angle of the liquid on the probe, L is the length of the rod and a is the radius.

The interfacial viscosity is calculated from the shearing velocity (Figure 9) of the magnetic needle (analogy with the rheology of the bulk).

(24) $$\;\;\;\;\dot{\varepsilon }\;=\;\frac{V}{h}\;$$

(25) $$\tau_{s}=\frac{F}{2L}\;$$

(26) $$\eta_{s}=\frac{\tau_{s}}{\dot{\varepsilon }}\;$$

The complex interfacial modulus is calculated as follows:

(27) $$\tau_{s}=\frac{F}{2L}=G{*}_{s}\;\frac{z}{R-a}\;$$

(28) $$G_{S}*=\frac{F}{z}\;\frac{R-a\;}{2L}=\frac{\tau_{s}}{\varepsilon_{0}}\;e^{i\tau (\omega )}\;=G^{\prime }\;(\omega )+iG^{\prime \prime }\;(\omega )$$

with

(29) $$G^{\prime }(\omega )=\left(\frac{\tau_{s}}{\varepsilon_{0}}).\cos(\delta \right)$$

and

(30) $$G^{\prime \prime }\;(\omega )=\left(\frac{\tau_{s}}{\varepsilon_{0}}).\sin\;(\delta \right)$$

where **F** is the amplitude of the total drag force exerted on the needle; **V**, **L** and a are respectively the velocity, the length of the needle and the radius of the needle; **R** is the distance between the axis of the needle and the semi-cylindrical glass cell; and **z** is the amplitude of displacement of the needle.

The magnetic needle rheometer can produce a very high Boussinesq number due to its low surface contact with the interface. If the geometrical factor is not sufficient to compensate for the drag forces of the sub-phases, corrections must be applied as explained above (Section 4.4). The algorithm is described in [36], and the implemented codes are available for download (https://softmat.mat.ethz.ch/opensource.html; last accessed 8 July 2022).

It is important to note that this technique is widely used in interfacial rheological studies, particularly in biology, to measure the surface (gas–liquid) tension and viscosity. This is the most sensitive technique among the interfacial shear measurements (the measured interfacial viscosity covers a wide range, from 10^−6^ to 10^−1^ Pa.s.m.); it is, however, more delicate to control because it is sometimes complicated to control the flotation of the needle, which relies on the surface tension (when the surface tension is too low, the needle sinks). This geometry is not appropriate for the study of interfacial rheological properties of viscous systems at low and high temperatures, such as polymer systems, because of their high viscosity and high melt temperature.

##### Pioneering Studies on the ISR

Based on the sensitivity of the ISR, there exist many studies that use this geometry to characterize interfaces. Here, we mention several studies on the ISR. The field of biology is the most studied with this geometry. Hermans and Vermant [43] focused on the study of the surface rheological properties of a phospholipid, DPPC (dipalmitoylphosphatidylcholine), on the air–water surface. They performed a complete temperature and surface pressure analysis of the interfacial shear rheology of DPPC, showing that the monolayer behaves as a viscoelastic liquid with a structure domain.

In another work [44], Hermans et al. studied the mechanisms affecting the stability of thin layers loaded with surfactants during their diffusion, using the drainage flows of a hemispherical dome. They concluded via the ISR that the presence of pulmonary surfactant replacement layers and DPPC monolayers at the air–fluid interface can improve the stability of thin films.

Gavranovic et al. [45] studied Langmuir monolayers of mixtures of straight and branched-chain hexadecanol forms using surface pressure isotherms on the ISR, at the air–water interface. The addition of branched molecules leads to a non-monotonic increase in surface viscosity.

Leiske et al. [46] studied the main components of the lipid layer of the tear film, referred to as the meibomian lipids. Using the ISR, they concluded that the interfacial meibum films were highly viscoelastic at 17 °C, but when the films were heated to 30 °C, the surface moduli decreased by more than two orders of magnitude.

Reynaert et al. [38] studied a purely viscous interface of known viscosity, produced by spreading thin silicon oil films onto a water layer, and a time-dependent viscoelastic interface was generated via the surface gelation of a lysozyme solution.

Freer et al. [47] studied protein adsorption at fluid–fluid interfaces after long-term exposure to produce interfacial gel-like networks. They used the ISR to examine the structure of a globular protein, lysozyme, and a disordered protein, α-casein, and to study the kinetics of the network formation at the hexadecane–water interface. For lysozyme, the shear moduli grow with interfacial aging, indicating a transition from fluid-like behavior at early stages to network formation (solid-like behavior). Conversely, the interfacial shear moduli of α-casein change very little with interfacial aging.

### 4.6. Interfacial Rotational Rheometers

The principle of the interfacial rotational rheometer is based on a two-dimensional Couette geometry, in which the rotor with a radius of **R_1_** rotates inside the stator with a radius of **R_2_** [48]. The following equations can be used to compute the interfacial viscosity:

(31) $$\tau_{int}=\frac{1}{4\pi }\;\frac{R_{2}{}^{2}-R_{1}{}^{2}\;}{R_{1}{}^{2}\;\;R_{2}{}^{2}}\;T$$

(32) $$\varepsilon_{int}=\frac{R_{2}{}^{2}+R_{1}{}^{2}\;}{R_{2}{}^{2}-\;R_{1}{}^{2}}\;\varphi \;$$

(33) $${\dot{\varepsilon }}_{int}=\frac{R_{2}{}^{2}+R_{1}{}^{2}\;}{R_{2}{}^{2}-\;R_{1}{}^{2}}\;\Omega \;$$

(34) $$\eta_{\mathrm{int}}=\frac{\tau_{int}}{{\dot{\varepsilon }}_{int}}\;$$

(35) $$\eta_{\mathrm{int}}=\;\frac{1}{4\pi }\;(\frac{1}{\;R_{1}{}^{2}}-\frac{1}{\;R_{2}{}^{2}}).\;\frac{T}{\Omega }\;$$

where **T**, **φ** and **Ω** respectively represent the torque applied to the rotor, the angle of rotation of the rotor and the angular velocity of the rotor. These equations are rigorously valid only for Newtonian liquids or in the case of a narrow air gap (**R_2_ − R_1_**)/**R_1_** << 1 (sufficiently high **Bo**).

#### 4.6.1. Du Noüy Ring

**Figure 10:**
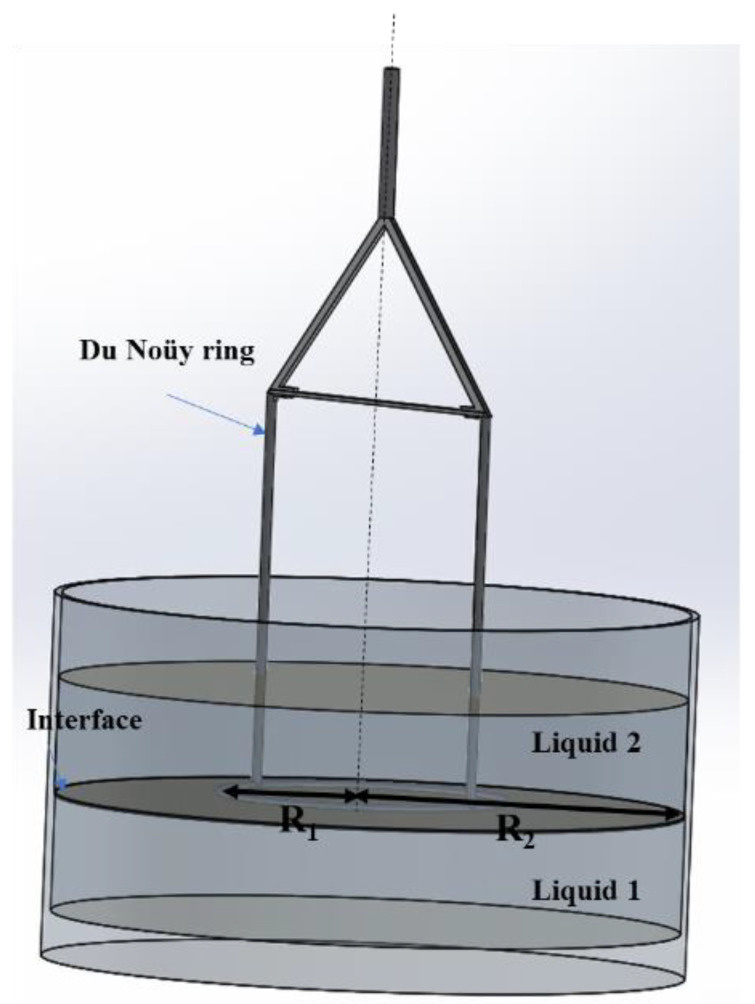
Profile section of the Du Noüy ring device.

In the beginning, this method was used for measuring the surface tension of a liquid. It is based on the same principle as the Wilhelmy plate method. Then, it was used for interfacial rheological measurements [49]. A horizontal ring is placed at the interface (or on the surface) and rotates or oscillates around its axis of rotation. This ring has a circular section, and a schematic profile of this geometry is displayed in Figure 10.

To measure the shear viscoelastic modulus, the applied equations are similar to the equations corresponding to the bidimensional cylindrical Couette geometry (they can be applied directly). The interfacial viscosity can be calculated by using the previous formulas of ***η*_int_** (Equation (35)).

Noting that **R_1_** is the radius of the ring and **R_2_** is the radius of the cup containing the sub-phases, M and **Ω** respectively represent the torque applied to the rotor and the angular velocity of the rotor.

In this case, the Boussinesq number can be expressed as:

(36) $$\mathrm{Bo}=\;\frac{\eta_{\mathrm{int}}}{\eta_{1}+\eta_{2}}\frac{P}{A}=\frac{\eta_{\mathrm{int}}}{\eta_{1}+\eta_{2}}\frac{\left({4\pi R}_{1}\right)}{\left({2\pi }^{2}R_{1}D\right)}=\frac{\eta_{\mathrm{int}}}{\eta_{1}+\eta_{2}}\frac{2}{\left(\pi D\right)}\;$$

where **D** is the diameter of the wire of the ring (0.36 mm), and ***ɳ*_1_** and ***ɳ*_2_** are respectively the viscosities of sub-phases 1 and 2.

The Du Noüy ring has a very light geometry with a low moment of inertia, which allows the measurement of interfacial properties. Furthermore, the geometrical factor 2/**πD** is very high, so the surface contact with the interface is low, leading to a high Boussinesq number. Nevertheless, if the geometrical factor is not enough to compensate for the drag forces of sub-phases (in other words, when **Bo** << 1), a numerical correction must be made to consider the effects of the sub-phases [36].

Despite this advantage, the Du Noüy ring also has some disadvantages, such as its fragility. It is easy to twist it during measurement (parts of the ring will be inside a sub-phase and not within the interface), which will lead to an erroneous calculation. Therefore, it is unsuitable for characterizing rigid interfaces (high interfacial viscosity ***η*s**) or high-viscosity sub-phases (for instance, polymers). Another inconvenience of this geometry is the way the Du Noüy ring is placed in the interface. This is related to its low contact area with the interface and its wetting properties (square section). Hence, the variation of the normal force is undetectable [49].

#### 4.6.2. Bicone

**Figure 11:**
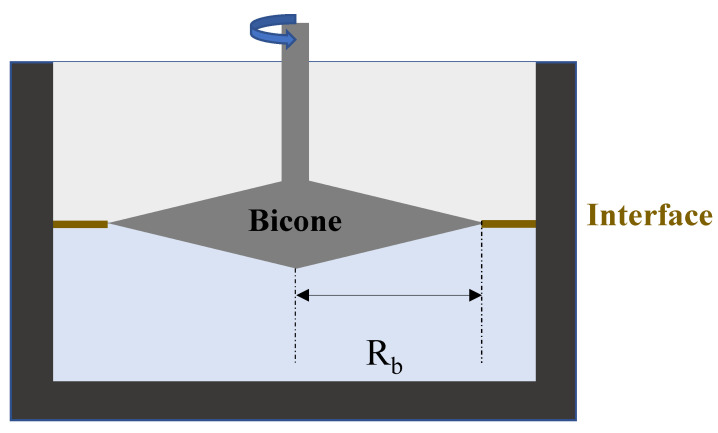
Profile section of the bicone device.

The bicone [40] is a robust geometry used to probe surfaces and interfaces. It is a two-dimensional projection of the Couette geometry used in bulk rheology. It is significantly tapered at its end (Figure 11). The densest liquid is poured first, and then the bicone is placed at the surface.

The edges of the bicone need to be located exactly at the interface between the two immiscible liquids. This geometry is marketed by TA Instruments and Anton Paar. It can easily be attached to a rheometer and performs both the steady and oscillating tests. The bicone Boussinesq number is expressed as:

(37) $$\mathrm{Bo}=\;\frac{\eta_{int}}{\eta_{1}+\eta_{2}}\frac{1}{R_{b}}\;$$

where **R_b_** is the radius of the bicone.

Independently of the ratio of the viscosities ($\frac{\eta_{\mathrm{int}}}{\eta_{1}+\eta_{2}}$) of the studied system, the value of the Boussinesq number will decrease due to the geometrical factor $(\frac{1}{R_{b}}$) being low. This geometry is not often used to characterize fragile interfaces (with very low interfacial viscosity) and is rarely used for viscous sub-phases because the drag forces produced on the bicone are sometimes non-negligible.

When **Bo** << 1, a numerical correction based on an iterative method of the apparent data needs to be made to consider the effects of the sub-phases and to define the corrected value of the measured torque T_c_ (see the previous expressions). The implemented routine is available for download (https://data.mendeley.com/datasets/4tmy9k4ys3/1; last accessed 8 July 2022).

Soo-Gun and Slattery [48] demonstrated an exact solution of the velocity profiles in the two sub-phases and at the interface. The authors made assumptions for the sub-phases as well as for the interface. First, they supposed that the mass and momentum were conserved for the sub-phases. Second, they assumed that there was a jump in mass balance at the interface, i.e., that the velocity was continuous at the interface, which means that the dynamics of each of the two fluids were driven by the continuity and Navier−Stokes equations.

(38) $$\mathrm{div}\;v_{\theta }=0\;\mathrm{with}\;v_{\theta }={\bar{V}}_{s}\;(\mathrm{in}\;\mathrm{the}\;\mathrm{above}\;\mathrm{Equation}(6))$$

where **v_θ_** is the interfacial velocity vector.

Moreover, the authors defined a jump in momentum balance at the interface; in other words, the balance between the pressure and bulk viscous stress jumps through the interface, the stress generated by the interfacial viscosity and elasticity and the stress resulting from the dynamic interfacial tension, which includes the equilibrium interfacial tension, the Marangoni effect and Gibbs elasticity.

**τ_1_.n_1_ + τ_2_.n_2_ + div(τ) +ρ_s_.g = 0** (39)

where **τ** is the interfacial stress tensor given in the Scriven–Boussinesq equation (Equation (6)), **n_1_** and **n_2_** are the normal vectors perpendicular to the interface pointing into phases 1 and 2, **ρ_s_** is the mass density of the interface and g is the gravity per unit mass.

Finally, Soo-Gun and Slattery imposed a few boundary conditions. (a) Both of the sub-phases, as well as the interface, are incompressible and Newtonian. (b) The velocity of the sub-phases on the top, bottom and side of the walls is zero, while at the edge of the rotating bicone it is equal to the velocity of the bicone. (c) Supposing that the interface is flat, the shape and velocity distribution of the interface are stationary (steady flow). (d) The interfacial deflection is ignored. Therefore, the usual stress jump does not involve Laplace pressure. The research assumes a low Reynolds number and neglects the secondary flows. (e) The interfacial mass balance simplifies the surface divergence term. There are no Marangoni effects in the tangential stress balance.

**Figure 12:**
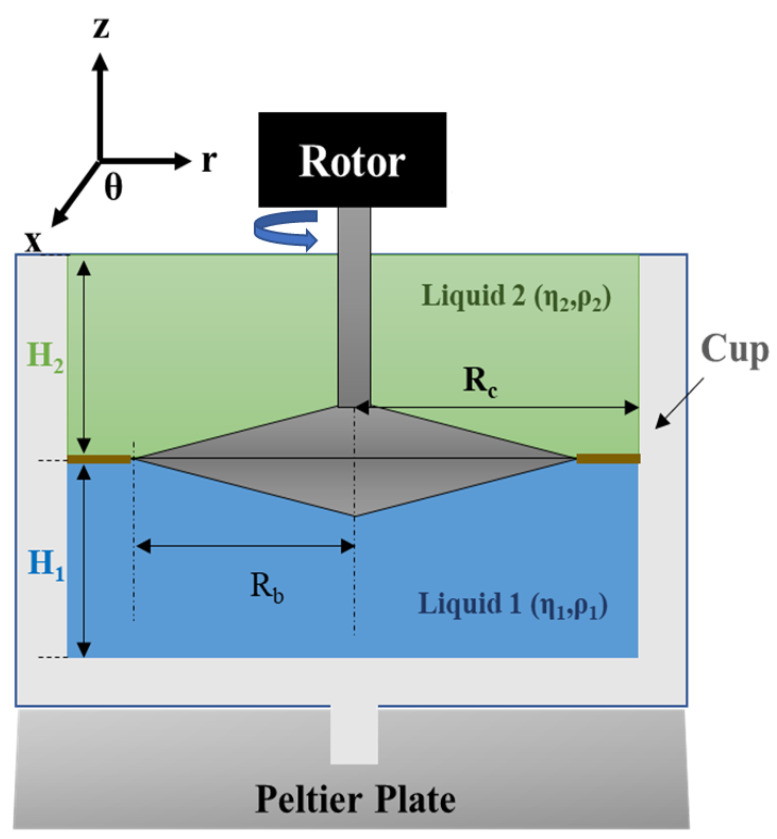
Schematic diagram of the rotating biconical bob rheometer [50]. Copyright 2022 Copyright AIP Publishing.

Once this distribution of velocities is known, the reduced torque ($\bar{T})\;$exerted by both liquids on the bicone and the interface (Figure 12) is determined according to:

(40) $$\bar{T}=\;\bar{R_{c}}3\;\mathrm{Bo}\frac{\partial }{\partial \bar{r}}{\left(\frac{\bar{v_{\theta }}}{\bar{r}}\right)|}_{\bar{r}=\bar{R_{c}}}-\int_{0}^{\bar{R_{c}}}{\frac{\partial {\bar{v}}_{\theta }^{1}}{\partial \bar{z}}|}_{\bar{z}=\bar{H_{1}}}\bar{r2}\;\;\mathrm{dr}+\frac{1\;}{Y}\;\;\int_{0}^{\bar{R_{c}}}{\frac{\partial {\bar{v}}_{\theta }^{2}}{\partial \bar{z}}|}_{\bar{z}=\bar{H_{1}}}\bar{r2}\;\bar{r}\;dr\;$$

(41) $$Y=\frac{\eta_{1}}{\eta_{2}};\;\bar{r\;\;}=\;\frac{r}{R_{1}};\;\bar{z}\;=\;\frac{z}{R_{1}};\;\bar{H_{1}}\;=\;\frac{H_{1}}{R_{1}};\;{\bar{v}}_{\theta }^{j}\;\;=\;\frac{v_{\theta }^{j}\;}{R_{1}\Omega -\bar{r}}\;$$

The exact solution of the reduced torque $\bar{T}\;$proposed by Soo-Gun and Slattery is:

(42) $$\bar{T}\;=\frac{T}{2\pi \Omega R_{b}{}^{3}\left(\eta_{1}+\eta_{2}\right)\;}\;$$

In this case, **T** is replaced by $\bar{T}$ in the expression of the interfacial viscosity (Equation (30)).

(43) $$\eta_{\mathrm{int},\mathrm{corr}}\;=\;\;\frac{1}{4\pi }(\frac{1}{R_{b}2}-\frac{1}{R_{c}2}).\;\frac{\bar{T}}{\Omega }\;$$

**Figure 13:**
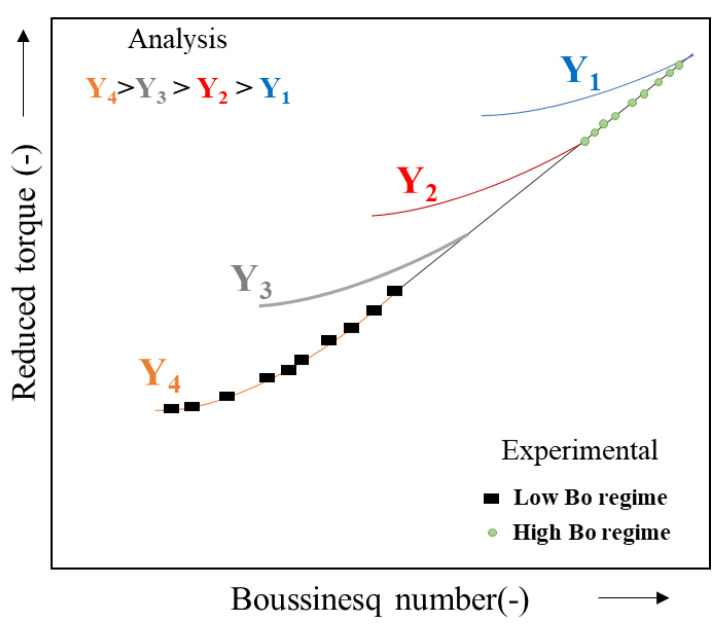
Reduced torque evolution as a function of the Boussinesq number at different bulk viscosity ratios [50]. Copyright 2022 Copyright AIP Publishing.

Soo-Gun and Slattery [48] have shown that the graphs of reduced torque as a function of Bo (Figure 13) are represented by curves that depend on the ratio of the volume viscosities. These curves all converge toward the same linear asymptote for high values of Bo according to the following relation:

(44) $$\bar{T}=\;\frac{2R_{b}2}{R_{c}2-R_{b}2}\;\mathrm{Bo}$$

(Line equation when **Bo** >> 1).

When the studied interface is rigid and the Boussinesq number is high, the interfacial stress must be calculated when R_c_/H_1_ and R_c_/R_b_ tend toward 0. The interfacial viscosity will be expressed as:

(45) $$\eta_{\mathrm{int}}=\frac{T-\frac{8}{3}\;R_{c}{}^{3}\;\left(\eta_{1}+\eta_{2}\right)\;\Omega }{4\pi R_{c}{}^{2}\;\Omega \;}$$

It should be noted that interfacial rheology measurements are not straightforward, especially when performed at the rheometer’s torque limit. The biconical geometry has a high weight (high momentum inertia); after extracting the inertia of the geometry and correcting for the effects of the sub-phases, the measured interfacial torque is very low or sometimes non-measurable, which makes the bicone less sensitive in this case and leads to less information from the collected data, particularly in the oscillating test, where the inertia is much more significant compared to the steady test. For this purpose, it is often used to characterize the viscoelastic properties of rigid interfaces (high interfacial viscosity). Probing the interfaces between two viscous polymers requires the adoption of a rigid, sturdy geometry in order to avoid deformation due to the high mechanical resistance of the polymers. El Omari et al. [51] introduced a novel biconical geometry manufactured from titanium (Ti) instead of stainless steel. This titanium-based geometry is half as dense as its stainless-steel equivalent, decreasing the geometry inertia and making the geometry more sensitive. This measuring setup also allows interfacial rheology measurements up to 200 °C, and gives the possibility of studying a wide range of molten polymer systems.

##### Pioneering Studies on the Bicone

The bicone is infrequently used in interfacial rheology because of its inertia and its average sensitivity. Here, we highlight some examples of pioneering studies. In the field of biology, Erni et al. [50] studied steady shear and oscillatory experiments, as well as creep recovery and stress relaxation tests at both oil–water and air–water interfaces with the adsorbed films of a globular protein (ovalbumin) and spread films of a surfactant (sorbitan tristearate).

The authors used the bicone in another work [52] to study the shear rheology of condensed interfacial layers (sorbitan tristearate) at the air–water interface. They defined the viscoelastic regime before performing shear experiments to avoid the destruction of the sample. By combining the interfacial rheology with microscopy, they concluded that interfacial films are both viscoelastic and fragile and are subject to fracture under small deformations, as shown by Brewster’s angular microscopy method performed in situ in rheological experiments to record surface morphology.

Ruhs et al. [53] studied the interfacial shear rheology of three systems: β-lactoglobulin fibrils, peptides and monomers adsorbed at the water–MCT (medium-chain triglycerides) interface at pH 2 through a time sweep at constant frequency and deformation. The amplitude sweeps were performed after 16 h, and no differences between these systems could be observed at pH 2. The interfacial elastic and viscous moduli increased when the pH increased.

It is useful to emphasize that this geometry can be efficient for probing rigid interfaces. Fan et al. [54] used the biconical geometry to study the interfacial shear rheology of asphaltenes (molecular substances that are found in crude oil) at the oil–water interface and their relation to the emulsion stability. They obtained a rigid interfacial film with a cross-linked structure due to the dispersion of the asphaltenes above a critical concentration in 20 h.

Ligiero et al. [55] studied the stability of oil–water emulsions by examining the interfacial films of surfactants already present in the crude oil. The authors used the bicone cell to define the shear interfacial rheological properties. They concluded that a perfect correlation with the emulsion stability and stronger mechanical properties lead to a more stable water–oil emulsion.

El Omari et al. [51] used a new bicone to study the interfacial properties at the interface of the two model fluids, polydimethylsiloxane (PDMS) and polyisobutylene (PIB). The authors also probed the interface of the immiscible molten polymers polycaprolactone (PCL) and polyethylene glycol (PEG). The apparent interfacial shear properties were measured in both oscillatory and steady flow modes. The contribution of the bulk sub-phases was corrected during the processing of the data, and the effects of the temperature and the molecular weight were also highlighted.

#### 4.6.3. Double-Wall Ring (DWR)

**Figure 14:**
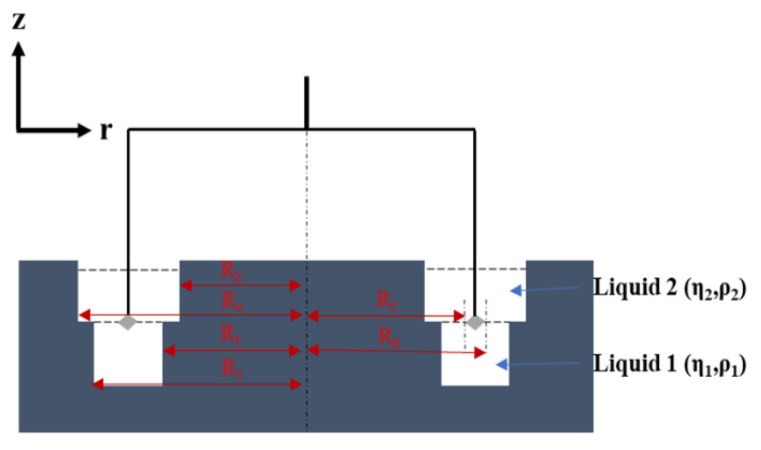
Schematic diagram of the double-wall ring (DWR) device.

The double-wall ring (DWR) combines the advantages of the Du Noüy ring, which is lightweight (less inertia), and the bicone (the contact area is more important than the Du Noüy ring). It is a geometry developed by TA Instruments [35]. Thus, just like the double-gap cylindrical Couette geometry, the double-wall ring has two gaps in which the interface is sheared (Figure 14).

**Figure 15:**
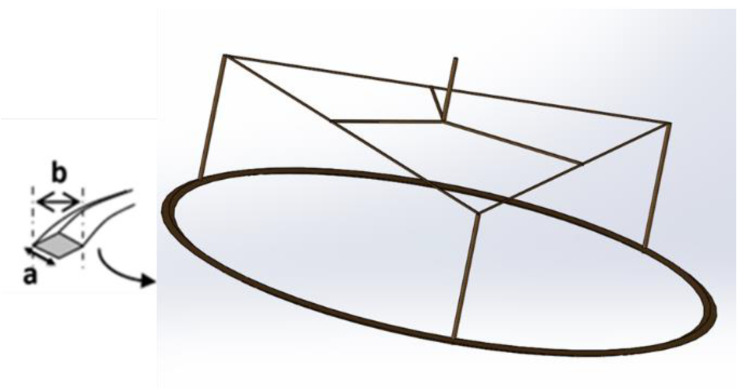
Schematic diagram of the square section of the ring (DWR).

It consists of a thin ring and a cup (in Delrin^®^, Teflon or metal) with a rounded channel. The segment of the ring resembles a square (with a precise edge in contact with the interface (Figure 15)) and is denoted a, which allows a better “grip” of the interface to the ring, in contrast to the rounded segment in the Du Noüy ring.

In order to benefit from the chemical inertness as well as the ease of cleaning and wettability, the ring and support feet are made of a platinum/iridium (Pt/Ir) material.

The double-wall ring (DWR) geometry is used to characterize interfacial thin films at both the gas–liquid surfaces and liquid–liquid interfaces. It helps to measure viscous as well as viscoelastic interfaces in the continuous shear and oscillatory experiments. The DWR can be attached to two types of rheometer: a CMT (combined motor transducer) and an SMT (separate motor transducer). The ring rotates or oscillates on the CMT rheometer, whereas on the SMT rheometer the cup rotates or oscillates [56].

In this case, the expression of the Boussinesq number is:

(46) $$\mathrm{Bo}=\;\frac{\eta_{int}}{\eta_{1}+\eta_{2}}\;\frac{1}{a}\;$$

where a is the diameter of the wire of the ring.

The interfacial stress can be expressed as:

(47) $$\tau_{\mathrm{int}}=\frac{T}{2\pi \left(R_{5}^{2}+R_{6}^{2}\right)\;}\;$$

The rate of interfacial deformation is given by:

(48) $${\dot{\varepsilon }}_{\mathrm{int}}=\left[\frac{1}{{\left(\frac{R_{5}}{R_{1}}\right)}^{2}-1}+\frac{1}{1-{\left(\frac{R_{6}}{R_{1}}\right)}^{2}}\right]\Omega \;$$

The expression of the interfacial viscosity using the DWR is:

(49) $$\eta_{\mathrm{int}}\;=\frac{T}{2\Omega \;\pi \left(R_{5}2+R_{6}2\right)\left[\frac{1}{{\left(\frac{R_{5}}{R_{1}}\right)}^{2}-1}+\frac{1}{1-{\left(\frac{R_{6}}{R_{1}\;}\right)}^{2}}\right]}\;$$

The geometrical factor $\frac{1}{a}$ is beneficial for obtaining a high Boussinesq number. If the Bo is low, i.e., if the interfaces are sensitive (when the interfacial viscosity is low), numerical corrections can be carried out. The velocity profiles in the sub-phases can be determined using the Navier–Stokes equation in the cylindrical coordinates (r, θ, z). The boundary conditions [35] are listed below.

The no-slip condition at the inner and outer walls of the DWR:

**v_1_(R_1_) = v_2_(R_2_) = v_1_(R_3_) = v_2_(R_4_) = 0** (50)

The no-slip condition at the moving ring surface:

**v_j_ (R_5_) = R_5_.Ω** (51)

**v_j_ (R_6_) = R_6_.Ω** (52)

Moreover, the stress condition at the interface is ruled by the balance of the tangential stress (Boussinesq–Scriven equation) at the interface (see Equation (19)).

As mentioned previously, the determination of the corrected interfacial viscosity is based on an iterative approach using the numerical computation of the velocity profiles (see Equation (13)) [35]. In this context, the expression of the calculated torque is given by:

(53) $$\mathrm{Tc}=2.п.\eta_{\mathrm{int}}.R_{5}^{3}\;\frac{d}{dr}\left(\frac{v_{int}}{r}\right)|r=R_{5}\;-\;2.п.\eta_{\mathrm{int}}.R_{6}^{3}\;\frac{d}{dr}\left(\frac{v_{s}}{r}\right)|r=R_{5}\;-\;2.п.\eta_{1}.\int_{R_{5}}^{R_{r}}\frac{dv_{1}}{dp_{1}}\;r_{2}.\mathrm{dr}\;\;-\;2.п.\eta_{1}.\int_{R_{r}}^{R_{6}}\frac{dv_{1}}{dp2}\;r_{2}.\mathrm{dr}\;-\;2.п.\eta_{2}\;.\int_{R_{5}}^{R_{r}}\frac{dv_{2}}{dp_{3}}\;r_{2}.\mathrm{dr}\;-\;2.п.\eta_{2}.\int_{R_{r}}^{R_{6}}\frac{dv_{2}}{dp_{4}}\;r_{2}.\mathrm{dr}\;$$

The algorithm is described in [36], and the implemented codes are available for download at https://softmat.mat.ethz.ch/opensource.html (last accessed 8 July 2022).

It should be noted that the DWR presents some disadvantages, such as the fragility of the ring. Moreover, the temperature range of the measurements is restricted because of the heating system, which is not thermally insulated (much of the heat is exchanged with the ambient air outside). To conclude, a large range of polymers cannot be studied with this technique; this includes polymers with a high melting point and high viscosity.

##### DWR and Controlled Surface Pressure

The double-wall ring can also be used for measurements under pressure [35]. In such cases, the measuring cell is modified; the idea is to merge the Langmuir balance (explained in the next section covering the interfacial dilatational rheology) and the DWR and to homogenize the pressure inside and outside the ring. The geometry of the ring is changed to achieve this kind of manipulation by adding three 5 mm openings to the ring (Figure 11 in [35]). The temperature can be controlled by a fluid bath. The surface pressure is controlled using a Wilhelmy plate in both the Langmuir trough and the DWR cup during the compression–expansion cycle.

##### DWR and Dilatational Measurements

In interfacial rheology, the shear and expansion measurement techniques are examined separately. Verwijlen et al. [57] designed a new geometry by modifying the DWR cell to make it sinusoidal, referring to it as the DWSR (Figure 1 in reference [57]), to simultaneously monitor both the expansion and the shear of the interface based on the following constitutive equation:

**τ_s_ = [σ + (*η*_d_ −*η*_s_)div_s_.v_s_ + (K − G) div.u_s_]I + 2*η*_s_D_s_ + 2GU_s_** (54)

where **u_s_** is the surface displacement vector (m), **U_s_** is the surface strain tensor for small deformation, and **K** and **G** are respectively the surface dilatational elasticity and shear elasticity (Pa.m).

This equation adds an elastic contribution to the Boussinesq–Scriven constitutive law. Due to the design of this geometry, the surface can be exposed and sheared, leading to a mixed flow. The elastic effect can be determined from the torque measurements.

In order to measure the complex dilatational interfacial viscosity from the torque measurements, a highly sensitive rotational rheometer is required, as well as a complex fluid interface whose torque is measurable in the sensitive region, with sensitivity values |Θ| between 20 and 2000.

The limitation of the torque is mainly affected by the shear surface viscosity, and the measured values are affected very little by interfacial rheology during dilatation (they are about 25 times more affected by interfacial shear). It should be noted that the interpretation of the raw data requires knowledge of linear interfacial shear rheology as well as a high Boussinesq number. In other words, it is necessary to perform a manipulation of the interfacial shear rheology with the different geometries we previously explained (DWR, ISR, bicone) before interpreting the DWSR raw data.

##### Pioneering Studies on the DWR

Vandebril et al. [35] were the first scientists who tried to study interfaces using a DWR cell. They probed the behavior of complex interfaces such as the interface of poly(octadecylmethacrylate) (PODMA)–water, an interfacial layer of lysozyme (protein) on the air–water surface and a commercial lung surfactant on the air–water surface.

In another application, the DWR was also used to study the role of the interface in coalescence in polymer blends using nanoparticles as compatibilizing agents. Vandebril et al. [3] demonstrated that the nanoparticles mainly affect the surface rheological properties between an immiscible system, e.g., polydimethylsiloxane (PDMS)–polyisobutylene (PIB). Based on the frequency sweep at a fixed strain on a layer of an ellipsoidal particle at the PIB–PDMS interface, they concluded that increasing the particle volume fraction suppresses the coalescence more efficiently. For highly loaded polymer blends, the microstructure even becomes independent of the shear rate and no coalescence happens.

One of the fields most studied using the DWR is biology. For instance, Sharma et al. [58] combined bulk and interfacial rheology to study the behavior of surfactant-free bovine serum albumin (BSA) solutions. They used the interfacial flow sweep to demonstrate that the origin of this yield-like behavior, which is manifested as a highly shear-thinning bulk rheological response, lies in the formation of a film of adsorbed protein formed at the solution–gas interface.

This conclusion was confirmed in another work by Jaishankar et al. [59] by comparing the interfacial rheological measurements with the corresponding interface; they demonstrated that the apparent yield strength presented by these solutions comes from the presence of a viscoelastic layer formed by the adsorption of protein molecules at the air–water interface.

Wang et al. [60] studied the behavior of the interfacial viscoelastic natural silk fibroin (protein) at both the air–water and oil–water interfaces. This natural multiblock copolymer forms a stable film at these interfaces. This interfacial layer presents a rheologically complex behavior with strong surface elastic moduli that is only slightly frequency-dependent.

The DWR has also been used in the study of foams and emulsions. Barman et al. [34] utilized the DWR to study the stability of foams and Pickering emulsions through the particle-laden interfaces (PLI) adsorbed on an air–water or oil–water interface. The bulk rheology and the stability of these foams and emulsions depend on the interfacial rheology of the PLI. This study required the correlation of the rheology with the microstructure by modifying the DWR cell to allow real-time, simultaneous interfacial visualization and shear rheology measurements. They demonstrated that the interfacial viscosity increases with increasing PLI concentrations and presents a shear thinning behavior.

Felix et al. [61] studied the dynamics of heterogeneous food products such as emulsions. These can be significantly affected by the interfacial properties of their interfaces. Proteins are widely used to increase the stability of these food products. This work compared the interfacial properties of a model protein (whey protein isolate, WPI) and silkworm pupae (SLW) adsorbed at the oil–water interface. A natural aldehyde (cinnamaldehyde, CNM) was used for both protein systems to promote protein–protein interactions. The results obtained from interfacial shear tests showed that the use of CNM resulted in the development of more robust interfaces, with higher values for the surface storage moduli and a lower loss tangent.

#### 4.6.4. Comparison between the Different Geometries

Here, we give a simple comparison between the geometrical factor that depends on the $\frac{1}{l}$ ratio of each geometry to give an idea of the Boussinesq number (Table 1):

## 5. Interfacial Dilatational Rheology

In contrast to the interfacial shear analysis, in interfacial dilational rheology, the test consists of measuring the surface or interfacial tension when the area of the interface changes [39]. In the expansion–compression tests, the viscoelastic properties of the interface are characterized by the 2D elastic modulus **E_d_** and the 2D dilatational viscosity ***η*_d_**:

(55) $$Ed=A_{0}\frac{d\gamma }{dA}\;=\frac{d\gamma }{dlnA}\;$$

and

(56) $$\eta d_{\;}=\frac{d\gamma }{dlnA/dt}$$

where **A_0_** is the initial interface area, **γ** is the surface tension and **A** is the interface area at time **t**. In the case of oscillatory measurements (harmonic testing of the interface at a frequency **ω**), a sinusoidal change in the surface tension can be observed with the presence of a phase shift. This can be explained by the viscoelastic nature of the interface. With low-amplitude surface deformation, the response of the interface (surface tension variation) is linear. It can be expressed as follows:

**γ = γ_0_ + Δγ sin (ωt + δ)** (57)

**A = A_0_ + ΔA sin (ωt)** (58)

where **ΔA** and **Δγ** are respectively the amplitude of the interfacial area and surface tension, while **A_0_** and **γ_0_** are respectively the interfacial area and surface tension just before the oscillatory deformation. In this case, we define the dilatational complex modulus E_d_* with a real part, the elastic modulus **E′**, presenting the stored and recoverable energy, and an imaginary part, the viscous modulus **E″**, referring to the energy dissipated by the system:

(59) $$E_{d}*=E^{\prime }+iE^{\prime \prime }=\left|E_{d}\right|\cos\delta +i\left|E_{d}\right|\sin\delta \;$$

Before performing a frequency sweep, it is mandatory to define the linear region. In other words, we need to define the critical deformation in order to study the viscoelastic properties of the surfaces (or interfaces) in their reversible equilibrium state without any change to their structure. Therefore, an amplitude sweep experiment at a specific frequency needs to first be performed. When the interfacial viscoelastic modulus begins to decrease at the highest strain amplitude, we refer to the non-linear regime.

**Figure 16:**
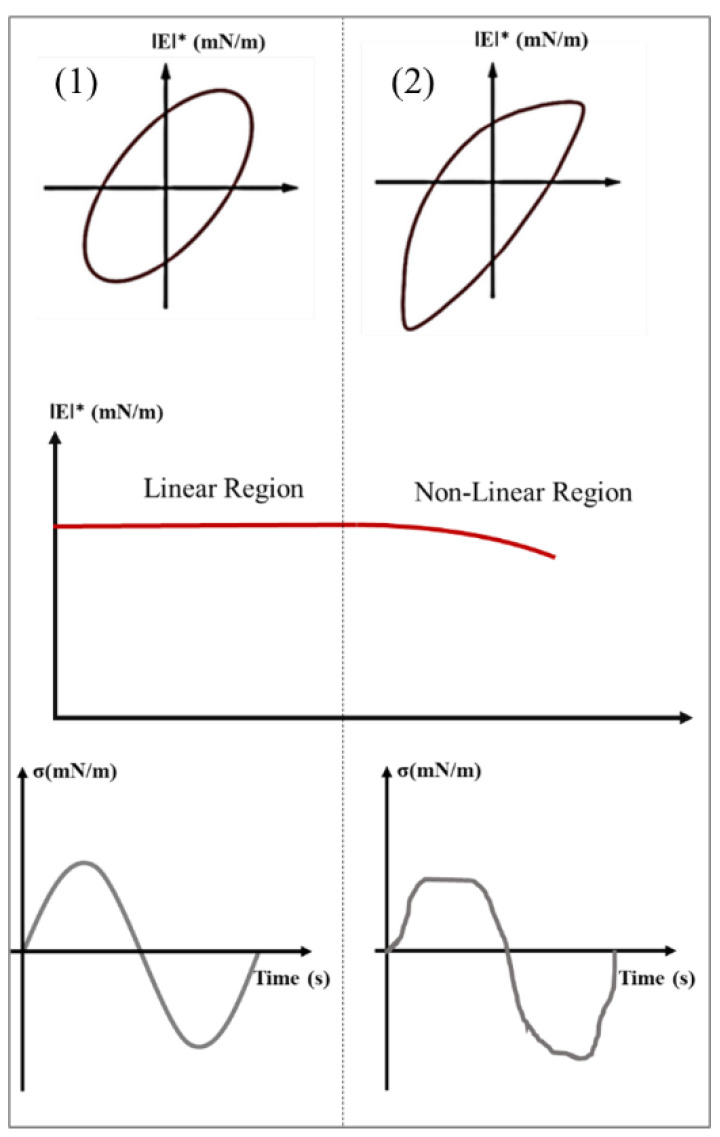
Lissajous plots in interfacial rheology: (1) the behavior in the linear domain and (2) the behavior in the non-linear region.

Another alternative to this classic strain sweep representation can be found in Lissajous–Bowditch plots (often referred to as Lissajous plots). This model was used extensively for bulk rheology and subsequently has been transposed for shear [62,63] and dilatational [63] deformations of the interface. Lissajous plots are graphically presented for surface (or interface) shear experiments by plotting the stress responses versus the shear rate or strain amplitude, and for dilational rheology by plotting the complex modulus E* or surface tension (or alternatively surface pressure) versus the deformation [64]. Figure 16 presents specific curves enabling the prediction of the linear (1) and non-linear (2) viscoelastic behaviors of the interfaces. In the case of linear dilatational interfacial rheology, the Lissajous plots are symmetrical, the variation of the dilatational modulus is independent of the applied strain and the variation of the interfacial tension follows a perfect sinusoidal curve, unlike the non-linear region.

Concerning the experimental part, there are two techniques that can be used to measure the interfacial dilational rheological properties. The first one is a direct measurement that consists of measuring the surface pressure (Langmuir balance). The second one is based on an optical contour analysis (oscillating pendant drop and spinning drop).

### 5.1. Langmuir Balance (or Langmuir Trough)

#### 5.1.1. Description

**Figure 17:**
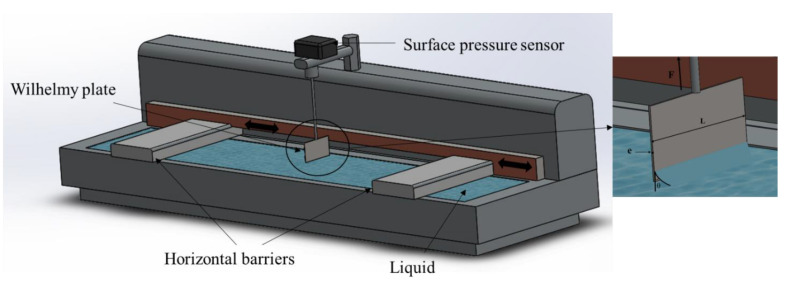
Schematic diagram of the Langmuir balance.

The Langmuir balance uses the Wilhelmy plate (Figure 17). The latter is a perfectly clean blade with a constant thickness e and length L (e << L) connected to a precise balance and immersed in a liquid. The liquid exerts an attractive force on the Wilhelmy plate, which depends on the nature of the liquid and the length of the blade [35].

When the Wilhelmy plate is immersed in a liquid, a meniscus is formed along the perimeter of the blade. The force exerted on the blade (blade pull-out force) is related to the surface tension by:

**F = P γ cosθ** (60)

where **P** is the perimeter of the blade, **γ** is the surface tension and **θ** is the contact angle.

The machine contains two barriers allowing the compression of the surface, while a pressure sensor above the tank allows the measurement of the surface pressure as a function of time and the area between the barriers. The two types of measurements are defined as described below.

#### 5.1.2. Compression at Constant Velocity Followed by Relaxation

Here, we measure the variation of the surface pressure:

**∆P = ∆Pe + ∆Pt** (61)

where **∆Pe** is the variation of the equilibrium pressure:

(62) $$∆Pe\;=E_{e}\frac{∆A}{A_{0}}$$

while **∆P_t_** is the transient pressure (dissipated energy):

(63) $$∆Pt_{\;}=E_{t}\;\frac{∆A.\tau }{A_{0}}(1-\exp(\frac{-t}{\tau }))\;$$

and **∆P_t_** is monitored as a function of time.

#### 5.1.3. Harmonic Stress (Oscillation) at a Frequency ω

**Figure 18:**
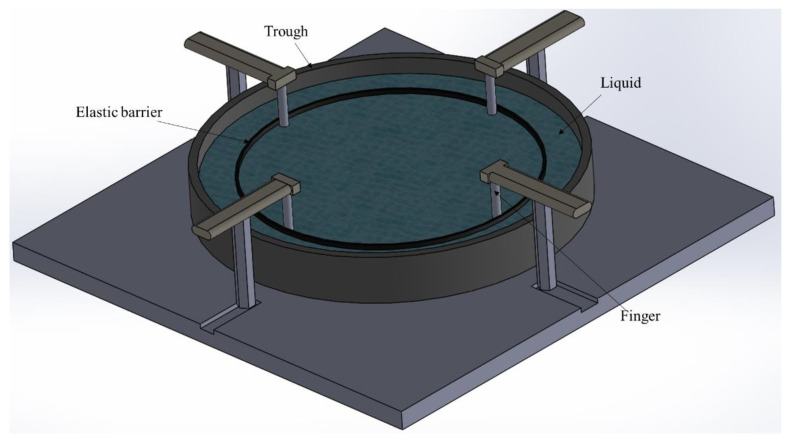
Schematic diagram of the radial trough setup composed of a circular Teflon trough with an elastic barrier and aluminum fingers, controlled by a stepper motor. Adapted from Ref. [27]. Copyright 1855 Copyright Taylor and Francis.

In the methods involving a Langmuir trough, the deformation is a combination of shear and dilatation. For this reason, Pepicelli et al. [27] designed another custom radial surface compression system, in which the deformation field is noticeably simpler, purely comprising dilation or compression. An elastic barrier is used to control the compression. Twelve fingers are used to apply uniform deformation to a radially shaped cup to facilitate a fluid mechanics analysis (Figure 18).

Note that the Langmuir balance allows an understanding of the dilatational rheological properties of surfaces of low-viscosity sub-phases. The design of this device does not permit measurements to be performed at high temperatures, and equipping this instrument with a heating system can help overcome this limitation.

### 5.2. Oscillating Drop Tensiometry

#### 5.2.1. Description of the Pendant Drop Method

**Figure 19:**
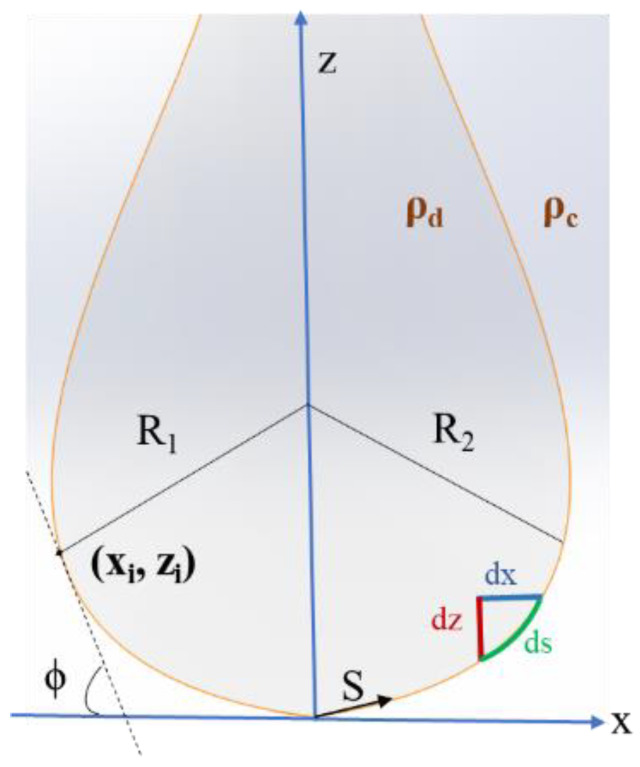
Schematic diagram of the characteristics of the pendant drop.

The oscillating drop tensiometry method is based on the study of the shape of a liquid (or gaseous) pendant drop (or a rising drop) at the end of a capillary within another liquid or gaseous phase (Figure 19).

The shape of the drop depends on the interfacial tension, which tends to make the drop spherical, and the difference between the density of the two fluids (gravitational effects), which tends to expand the drop and changes its spherical shape. The drop then takes on the form of a “bulb” [65].

In theoretical terms, at equilibrium, the drop (or bubble) obeys the Young–Laplace equation linking the Laplace pressure to both the radius of the curvature and the interfacial tension:

(64) $$\gamma \left(\frac{1}{R_{1}}+\frac{1}{R_{2}}\right)=∆P=∆P_{0}-∆\rho \mathrm{gz}\;$$

where **γ** is the surface (or interfacial) tension, **R_1_** and **R_2_** are respectively the first and second principal radii of curvature, and **∆P** = **P_in_** − **P_out_** is the Laplace pressure, which is the pressure difference across the interface.

Here, **∆P_0_** is a reference pressure at **z** = 0, whereas **∆ρgz** is the hydrostatic pressure;

**∆ρ** = **ρ_d_** − **ρ_c_** is the density difference; **ρ_d_** and **ρ_c_** are respectively the density of the drop phase and the density of the continuous phase.

**Figure 20:**
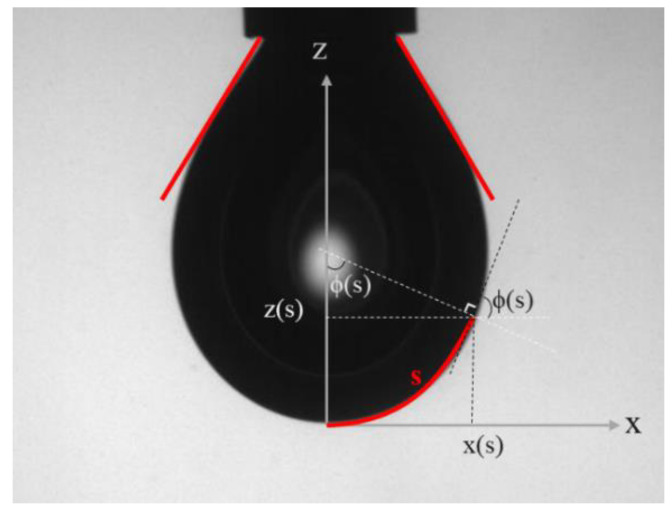
A pendant drop image indicating the coordinate system used for determining the surface tension. The drop shape is fit to a solution of the Young–Laplace equation.

By fitting the drop contour, the surface (or interfacial) tension can be extracted from the images of the drop (Figure 20).

Another important parameter that needs to be well defined is the Bond number Bd (dimensionless) [65]. It represents the ratio between the gravitational forces and the interfacial tension. The shape of the drop depends on this number:

(65) $$B_{d}\;\;=\frac{g∆\rho R_{app}^{2}}{\gamma }\;$$

The more the drop is spherical, the lower the Bond number becomes and the more the relative error of the measurement increases. By observing the expression of **B_d_**, and in order to increase the accuracy of the measurement, it is recommended to increase the Bond number by increasing the radius of curvature (increasing the volume of the drop) or by increasing the difference in densities of the coexisting phases [50].

The Young–Laplace equation can be expressed in cylindrical coordinates r and x, according to differential equations in terms of the arc length s measured from the drop’s apex, the Bond number Bd and the tangent angle **ϕ** in a curvilinear coordinate system of x(s) and z(s) (the smoothed solution of the Laplace equation).

The first and second principal radii of curvature are defined as:

(66) $$R_{1}=\frac{ds}{d\phi }\;\;\;\;$$

(67) $$R_{2}=\;\frac{x}{sin\phi }\;$$

Considering the axisymmetry of the drop or bubble, the curvature at the apex is constant in all directions:

(68) $$R_{\mathrm{apex}}=R_{1}=R_{2}\;$$

The reference pressure can be expressed as:

(69) $$∆P_{0}=\;\;\frac{2\gamma }{\;R_{apex}\;}$$

Therefore, Equation (64) can be obtained as a coupled set of differential equations in terms of the arc length x measured from the drop apex:

(70) $$\frac{d\phi }{ds}\;=2-B_{d}z-\frac{sin\phi }{x}\;$$

(71) $$\;\;\;\;\;\;\frac{dx}{ds}\;=\cos\phi \;$$

(72) $$\;\;\;\;\;\;\;\frac{dz}{ds}\;=\sin\phi \;$$

where **R_apex_** is the radius of curvature of the surface.

Here, a comment needs to be added. The fitted Young–Laplace solution can also be used to give additional data, such as the drop volume **V_d_** and drop surface area **A_d_**, which are defined as:

(73) $$Vd_{\;}=\pi \int r2sin\phi \;\mathrm{ds}\;$$

and:

(74) $$Ad_{\;}=2\pi \int r\mathrm{ds}$$

These two relationships allow the extraction of other interesting interfacial data. For example, the percentage of evaporation of the pendant drop can be quantitatively determined by monitoring the variation over time of the volume of the drop (or bubble) [66].

#### 5.2.2. Precision of the Pendant Drop Method: The Worthington Number

**Figure 21:**
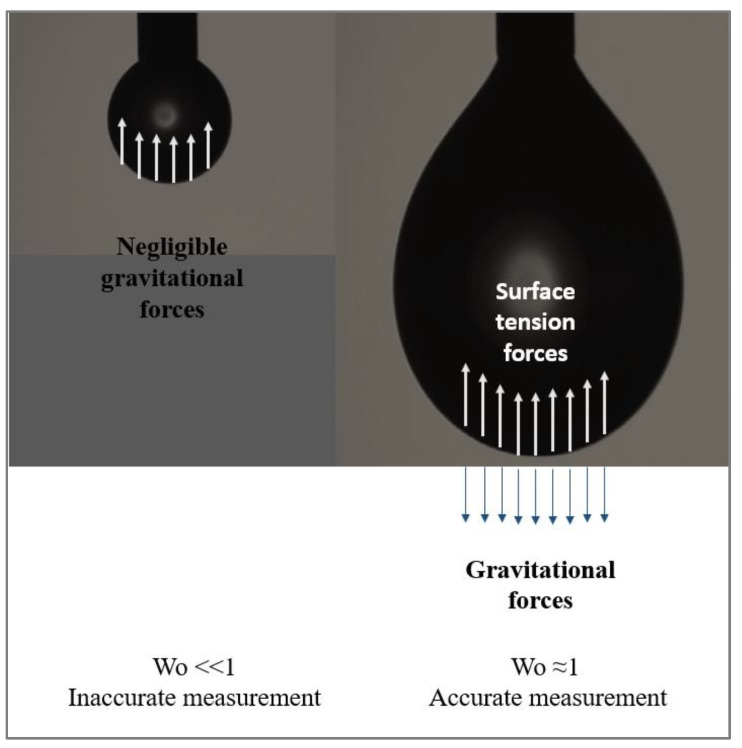
Accuracy of the pendant drop method.

According to the Bond number expression, it is interesting to note that as the diameter of the needle decreases, the minimum Bond number value needed for an accurate calculation decreases. Berry et al. [65] introduced a universal solution independent of the needle diameter by using a dimensionless number, which represents the ratio of the drop volume **V_d_** (calculated with Equation (73)) to the theoretical maximum drop volume **V_max_**. Berry et al. [65] referred to this parameter as the Worthington number **Wo** (Figure 21). It is expressed as follows:

(75) $$\mathrm{Wo}=\frac{V_{d}}{V_{max}}$$

where **V_max_** is expressed as:

(76) $$V\max_{\;}=\frac{\pi D_{n}\gamma }{∆\rho g}$$

and **D_n_** is the external diameter of the needle.

It should be noted that before launching the measurements, it is necessary to determine the volume corresponding to the detachment of the drop, **V_max_**.

#### 5.2.3. Rheological Measurements in the Harmonic Regime (Oscillation at a Given Frequency)

**Figure 22:**
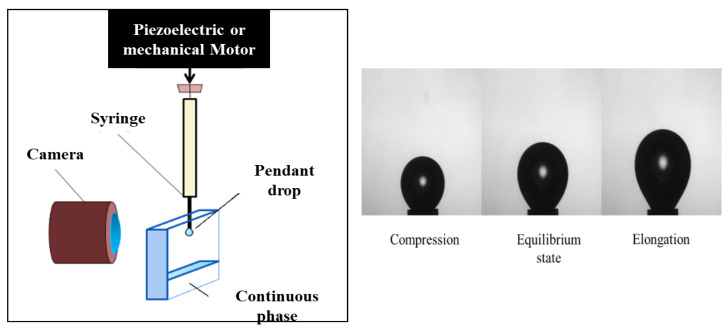
Close-up views of an oscillating drop.

**Figure 23:**
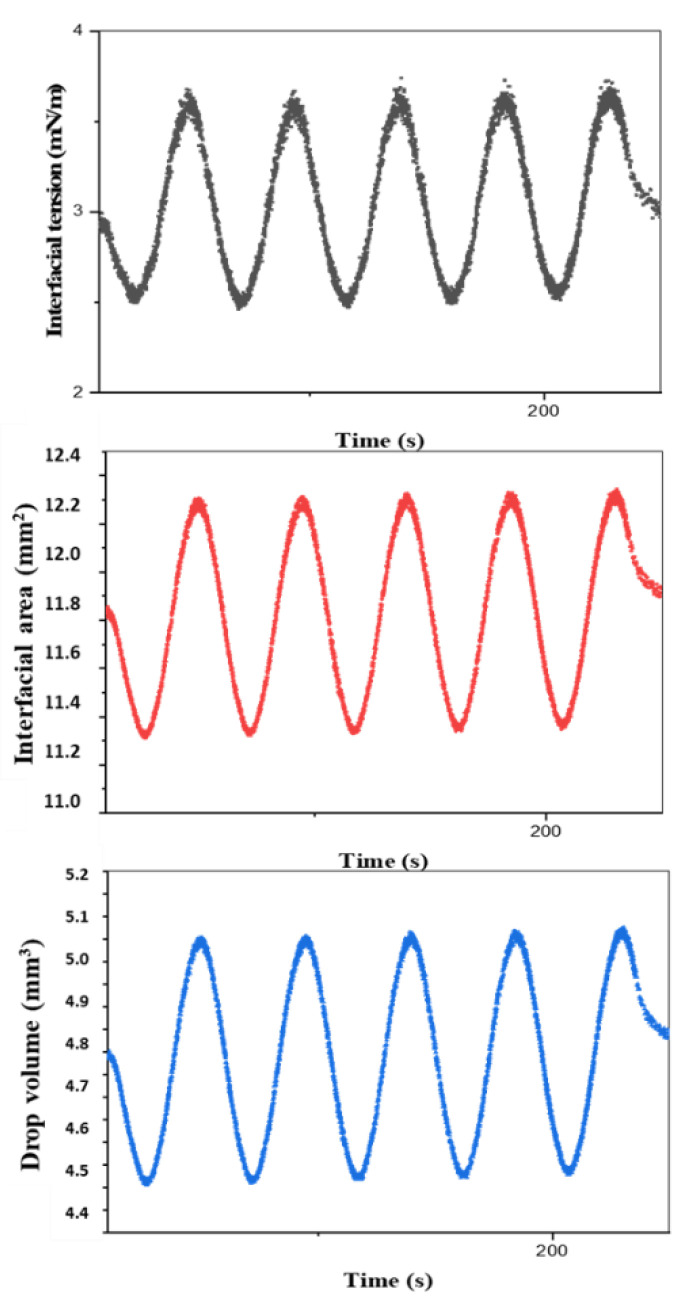
The interfacial tension (mN/m), surface area A (mm^2^) and drop volume V (mm^3^) versus time t (s).

The drop is subjected to a change in volume (through a piezoelectric or mechanical piston (Figure 22)) with a sinusoidal deformation profile (Figure 23), and the shape of the drop at each oscillation makes it possible to calculate the surface or interfacial tension via the Young–Laplace equation.

Since the pioneering articles that appeared in 1983 [67], developing the computational routines used for all available data of the pendant drop profile, which greatly increased the precision of the fitting methods, other papers have been published [65]; among these, we can cite the recent article by Kratz and Kierfeld [68].

#### 5.2.4. Influence of the Viscosity and Elasticity on the Interfacial Dilatational Measurements

##### Effect of the Continuous-Phase and Drop-Phase Viscosity

When measuring the static surface or interfacial tension with the pendant drop method, the gravitational effects acting on the drop or bubble are in equilibrium with the interfacial effects. In this case, the Young–Laplace equation is valid.

However, in the case of oscillatory measurements, other forces appear because of the change in the interfacial area during the experiment. In this instance, inertial and viscous forces modify the shape of the drop or bubble. Consequently, the Young–Laplace equation is invalid, and the measuring frequency range is restricted (the maximum frequency is limited) [69].

Other parameters need to be quantified in order to estimate the inertial and viscous forces [69]. They are the capillary number **Ca**, defined as the ratio of viscous to capillary forces, and the Weber number **We**, defined as the ratio of inertial to capillary forces:

(77) $$\mathrm{We}=\frac{\;∆\rho \omega 2∆V2}{\gamma R_{c}^{3}}$$

(78) $$\mathrm{Ca}=\frac{\;∆\eta \omega ∆V}{\gamma R_{c}2}\;$$

where **Δ*η*** and **Δρ** are respectively the differences between the Newtonian viscosities and the densities of the drop and the continuous fluid, **ω** is the oscillation frequency of the drop, **ΔV** is the amplitude of volume oscillation, **σ** is the equilibrium interfacial tension and Rc is the radius of the capillary.

In the limits of **Ca** << 1 and **We** << 1, the drop shape is that of a static drop and is well described by the Young–Laplace equation.

To determine the dilatational behavior of viscous systems where the inertia is important, the principle of the time–temperature superposition is used to artificially extend the pulsation gap at a given temperature. Bouriat et al. [70] demonstrated that for a wide range of pulsations between 0.001 and 10 rad/s, the log–log curve of the elastic modulus **E** (expansion test) is a straight line, while the loss angle stays constant. Chambon and Winter [71] showed that for a crosslinking polymer system near its gelation point, the loss and storage moduli are parallel at the gel point, with a pulsation dependence of:

**E′****∝****E″****∝****ω^n^** (79)

where 0 < **n** < 1 is a relaxation exponent. A gel with n approaching 1 is purely viscous, whereas for **n** approaching 0 it is purely elastic. The loss angle **δ** is related to **n** by:

(80) $$\delta =\;\frac{n\pi }{2}\;$$

Abi Chebel et al. [72] demonstrated that for moderately aged crude oil droplets (≤5000 s), the elasticity and viscosity at a high frequency (10−80 Hz) could be extrapolated from low-frequency measurements (≤1 Hz) by the same power law of the frequency, **ωn**.

##### Main Physical Contributions to the Measured Signal and Calculated Surface Rheological Parameters 

**Figure 24:**
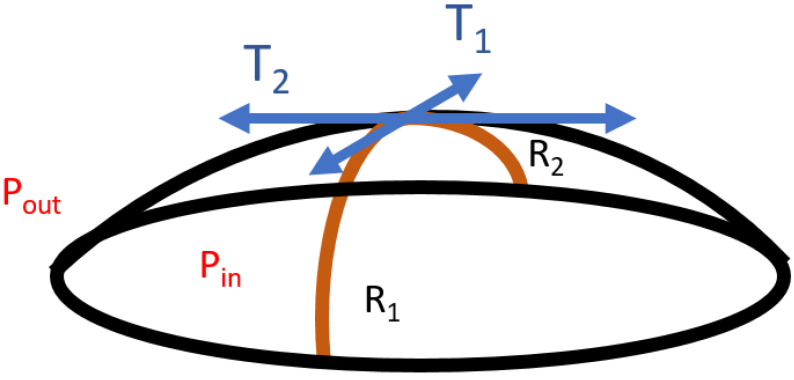
Representation of the parameters defining the interface.

Regardless of the nature of the interface, the Laplace equation can be written for each point of the interface as:

(81) $$∆P=P_{in}-P_{out}=\frac{T_{1}}{R_{1}}+\frac{T_{2}}{R_{2}}$$

where **T_1_** and **T_2_** are respectively the surface tensions in two perpendicular directions along the interface (Figure 24).

In static measurements, according to the Laplace equation, the surface (or interfacial) tension [73] varies linearly with the capillary pressure **Pc**. For reasons of symmetry, every direction is equivalent, so Equation (81) becomes:

(82) $$\gamma =\;\frac{P_{c}.R_{apex}}{2}\;$$

However, in the dynamic measurements, **P_app_**, the pressure applied to form the drop or bubble contains the capillary pressure (**P_c_**) in addition to other contributions, such as the hydrostatic pressure, **P_hydr_** (considered negligible), and **P_dyn_**, the dynamic pressure (due to the deformation of the drop volume) [70].

**P_app_ = P_hydr_ + P_dyn_ + P_c_** (83)

Note that in the static measurements, the dynamic pressure equals zero (mechanical equilibrium, where **P_c_** = function (γ) and **P_dyn_** = 0). In contrast to this case, during the oscillatory measurements, the capillary pressure is proportional not only to the surface tension but also to the viscous forces.

The complex interfacial modulus is obtained by replacing Equations (82) and (83) in the expression of the surface tension and generalizing Equation (8) from [74] to the following equation:

(84) $$E*=\;\frac{d\gamma }{d\;lnA}=\;(1\;-\;\frac{R_{s}{}_{,eq}\;}{H_{s,eq}\;\;})+\frac{R_{s,eq}}{2}\frac{d}{d\;lnA}(P{\mathrm{app}}_{\;}-\;P{\mathrm{hydr}}_{\;}-\;P_{\mathrm{dyn}})\;$$

where **H_s,eq_** and **R_s,eq_** are respectively the initial drop height and the initial radius (apex) before starting the oscillation (the equilibrium value). Equation (84) can be written in a revised form:

(85) $$E*={E*}_{0}\;-\;\frac{R_{s}{}_{,eq}\;}{H_{s,eq}\;}\frac{dP_{dyn}}{d\;lnA},$$

(86) $${E*}_{0}\;\equiv \;\gamma \;(1\;-\;\frac{R_{s}{}_{,eq}\;}{H_{s,eq}\;\;})+\frac{R_{s}{}_{,eq}}{2}\frac{d}{d\;lnA}(P_{\mathrm{app}}-P_{\mathrm{hydr}})\;$$

Viscous and inertial contributions are included in **P_dyn_**. For frequencies larger than 10 Hz, the inertial term in **P_dyn_** becomes more significant (the inertial forces are neglected by using frequencies lower than 10 Hz). Hence, the viscous contribution in **P_dyn_** consists of two parts.

**Figure 25:**
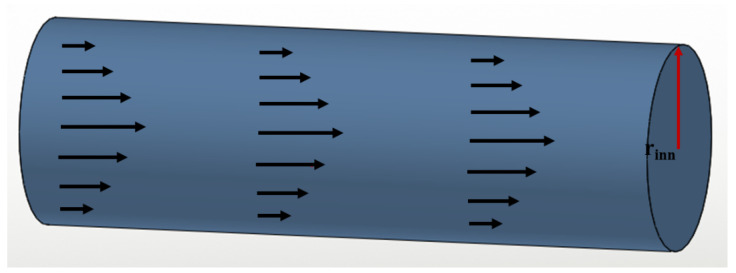
The flow of a Poiseuille tube inside a needle.

The first part is the Poiseuille pressure (**P_Pois_**) due to the flow in the capillary (Figure 25); for a Newtonian fluid, **P_Pois_** is given by the Poiseuille equation.

(87) $$P_{\mathrm{Pois}}=\;\frac{8\eta_{inn}L}{\pi r_{inn}^{4}}\;\frac{dV}{dt}=8\frac{R^{2}{}_{s,eq}H_{s,eq}\;L}{r_{inn}^{4}}\frac{dlnA}{dt}\;$$

where ***η*_inn_** is the dynamic viscosity of the drop phase moving inside the capillary, **L** and **r_inn_** are respectively the capillary length and its inner radius and **dV**/**dt** is the flow rate (the variation of the drop volume (cm^3^/s) **V**, as a function of time).

The second part of the viscous contribution in **P_dyn_** is the viscous pressure (**P_visc_**) due to the bulk viscous forces acting from both adjacent phases on the drop surface. Two different methods are used to compute the **P_visc_** [75]:

In the first place, by using the method of similarity and dimensional analyses in [76,77], the following relationship for **P_visc_** is obtained:

(88) $$P_{\mathrm{visc}}=\chi \;\frac{\eta_{out}}{r_{c}}\;\frac{dH_{s}}{dt}=\chi \;\frac{(\eta_{out}-\eta_{inn})R_{s,eq}\;}{r_{c}}\frac{dlnA}{dt}\;$$

where **r_c_** is the radius of the drop contact line, while the dimensionless parameter **χ** accounts for the drop geometry (**χ** should be a function of the ratio **χ** = **H_s_**/**r_c_**).

In the second place, by theoretically solving the hydrodynamic problem of a dilatational or compressive spherical drop attached to a capillary in an outer fluid phase in [77], the hydrodynamic drag force (**F_visc_**) can be calculated. When this force is applied in the circular section of the capillary, it creates pressure. This pressure can be expressed as follows:

(89) $$P_{\mathrm{visc}}=\frac{F_{visc}}{\pi r_{c}^{2}}\frac{\pi r_{inn}^{2}}{\pi r_{c}^{2}}=\frac{R^{2}{}_{s,eq}H_{s,eq}r_{inn}^{2}\;}{\pi r_{c}^{5}}[\eta_{inn}\mathrm{fa},0+\eta_{out}(\mathrm{fab}+\mathrm{fb})]\;\frac{dlnA}{dt}$$

As is obvious from Equations (88) and (89), the viscous stress contributions in **P_visc_** depend on the rate of the surface deformation (**dlnA_s_**/**dt**). They only contribute to the imaginary part of the complex interfacial modulus. Therefore, the expression of the dilatational elastic modulus (the real part of the complex modulus E*) (see Equation (86)) is:

**E′ = E′_0_** (90)

while the dilatational loss modulus can be presented as:

**E″ = E″_0_ − E″_Pois_ − E″_visc_** (91)

where **E″_Pois_** and **E″_visc_** are the dynamic corrections to the loss modulus due to the flow in the capillary and to the drop in dilatation or compression, respectively.

(92) $$E_{\mathrm{visc}}^{\prime \prime }=\chi \;\omega \frac{\left(\eta_{out}-\eta_{inn}\right)R2_{s,eq}\;}{r_{c}}\;$$

(93) $$E_{\mathrm{Pois}}^{\prime \prime }=\;4\omega \eta_{inn}\frac{R^{3}{}_{s,eq}H_{s,eq}\;L}{r_{inn}^{4}}\;$$

(94) $$E^{\prime \prime }=E_{0}^{\prime \prime }\;-\;\chi \;\omega \frac{\left(\eta_{out}-\eta_{inn}\right)R2_{s,eq}\;}{r_{c}}-4\omega \eta_{inn}\frac{R^{3}{}_{s,eq}H_{s,eq}\;L}{r_{inn}^{4}}\;$$

Equation (94) shows that it is necessary to carry out the above for each correction of the dilatational loss modulus through the subtraction of the viscous and inertial contributions.

#### 5.2.5. Effect of the Elasticity of the Interface

When the interface (or surface) is elastic, i.e., the interface can support a shear stress in the interfacial plane, the elasticity of the interface in the dilatational measurement is the sum of the interfacial tension γ and the interfacial elastic tensor. This is very often used in the study of interfacial membranes [78] when the variation in the surface shear modulus reflects changes in the thermophysical properties of the interfacial film or the number density (cross-link density) of intermolecular interactions at the interface [79]. A neo-Hookean model was used to model the small deformation of the interface in this instance. The interfacial elastic tensor is given in Equation (4) from [79].

As mentioned earlier, a drop shape analysis or pressure tensiometry is used to extract the surface–material properties of these complex interfaces. The use of the Young–Laplace equation needs to be generalized to account for the extra and anisotropic stresses at the interface [80]. Shape-fitting methods, when combined with an adequate constitutive approach, do not perform well in defining the elastic stresses, especially for small deformations. Nagel et al. [81] used an additional measurement of the pressure or capillary pressure tensiometry to improve the sensitivity. This requires knowledge of the undeformed reference state (when the interfacial elastic tensor is negligible), which may be challenging to achieve in practice.

#### 5.2.6. Operating Parameters That Can Disturb the Measurement

Many parameters can modify the measurements. Among these parameters, some may even disturb the measurements.

**Vibration:**

The first parameter that can disturb the measurements is the vibration due to the external environment (workplace insulation, sound, noise, etc.). To limit vibrations during the measurement, the box that contains the device should be equipped with rubber shock absorbers and placed on a stable support.

**Verticality of the needle:**

The symmetry of the drop and the balance of the forces exerted on the drop can be changed if the needle is not strictly vertical.

**Impurities:**

The presence of an impurity can disturb the measurements either by modifying the properties of the environment or by complicating the visualization of the drop, as well as the detection of the edges of the drop (or bubble). For example, air bubbles may be considered an impurity. To fix this problem, the samples should be degassed.

**Drop configuration:**

**Figure 26:**
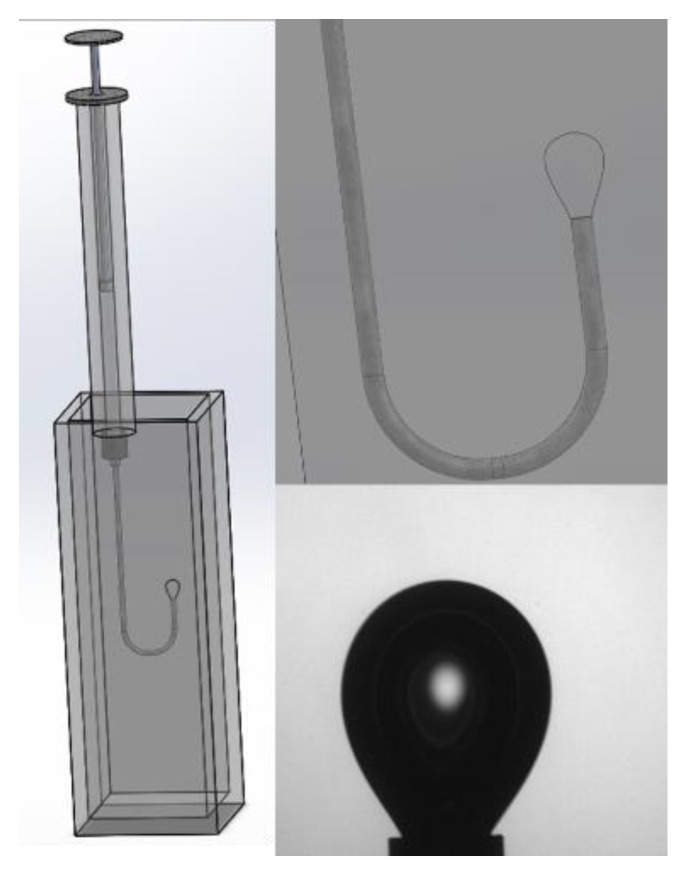
Rising drop formed upward at the tip of a U-bend.

If the value of **∆ρ** is positive (**∆ρ** > 0), we must use the pendant drop; if it is negative (**∆ρ** < 0), we must use the rising drop. In this case, the drop is formed upward at the tip of a U-bend (Figure 26).

**Stabilization time:**

First we form the drop, then the variation of the interfacial tension is monitored as a function of time until a constant value (plateau) is reached. Note that the experimental conditions should be constant, especially the parameters that most affect the interfacial (or surface) tension, such as the temperature and vibration.

Another issue that should be mentioned is the material characteristics, which could influence the accuracy of the measurement. For example, in the case of thermo-sensitive polymers, when the temperature increases and if the stabilization time to reach the equilibrium is long, the polymer may be degraded. Therefore, the measurements will be wrong. Taking this into consideration, the measurements should be made under an inert atmosphere.

**Some pioneering studies on dilational interfacial rheology based on the oscillating drop method**

Ruhs et al. [77] performed oscillatory surface amplitude measurements with a pendant drop tensiometer to determine the linear regime of β-lactoglobulin fibrils and their derivatives. The deformation experiments combined with a frequency sweep of the interfacial films showed different surface rheological behaviors at high and low frequencies. These observed dilatational rheological responses were described using two different adsorption models, the Maxwell model and a modified Lucassen and van den Temple model.

Rühs et al. studied in another work [53] the static and dilatational responses of β-lactoglobulin fibrils and native β-lactoglobulin (monomers) at the water–air and water–oil interfaces at pH 2 using the pendant drop method. The resulting adsorption behavior and the viscoelasticity were dependent on the concentration and adsorption time. They observed that with higher concentrations, they achieved fast adsorption kinetics and slightly higher interfacial and surface pressures, but this did not lead to higher viscoelastic moduli.

Understanding the properties and behaviors of diluted bitumen–water interfaces under stresses is essential to find a solution to emulsion problems in bitumen production. Angle and Hua. [82] studied the interfacial dilational rheology of toluene-diluted mineral-free Athabasca bitumen. For all oscillation frequencies studied, they observed that the interfacial viscoelastic modulus of Athabasca bitumen in toluene reached a maximum at 0.865 g/L (0.1 wt%) bitumen, then declined as the bitumen concentration increased.

A recent work by Gimenez et al. [83] focused on escin (a natural mixture of triterpenoid saponins isolated from horse chestnut seeds). The stabilized air–water interfaces displayed a highly non-linear behavior during dilatation, as evidenced by the Lissajous plot method. The use of surface rheological measurements beyond the commonly measured linear domain has provided new insights into the behavior of these interfaces and their microstructure.

The oscillating pendant (or rising) drop is also present in the study of oil extraction. Ligiero et al. [73] explored the characterization of a crude oil interfacial film (IM) isolated from wet silica via the oscillating rising drop method at 30 °C. Dilatational rheology measurements revealed that successive IM extracts from crude oil are composed of molecules that behave increasingly like insoluble surfactants that aggregate at the water–oil interface. These surfactants would be responsible for hindering the mechanical characteristics of the interface.

Abi-Chebel et al. [72] evaluated the dilatational rheology of a pendant-shaped diluted crude oil within water with an interface that contained a significant amount of surfactant. At a low frequency, the fluid viscosity and inertia were negligible, which allowed a direct determination of the dilatational interfacial rheology. This interfacial film underwent a viscoelastic behavior, with the elastic moduli being larger than the viscous moduli.

### 5.3. Spinning Drop Method

**Figure 27:**
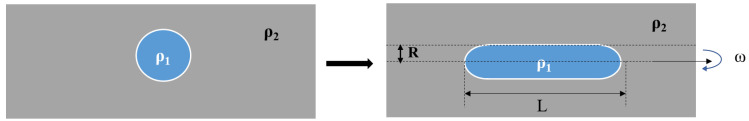
Schematic diagram of the spinning drop principle.

Based on the same principle as the pendant drop method, spinning drop tensiometry uses the optical contour analysis of a drop to determine the minimal interfacial tensions (below 1 mN/m), which cannot be performed with the other interfacial tension devices. Instead of dosing a drop using a syringe and a needle, a drop of the less dense liquid is put inside another liquid of higher density. The drop is exposed to gravity and placed inside a rotating capillary tube. Due to the centrifugal forces, the drop is deformed cylindrically toward the rotational axis (Figure 27) and its interfacial area increases. The interfacial tension counteracts this increase in area and can, thus, be determined by analyzing the shape of the drop in the equilibrium state.

This method was developed by Vonnegut [84]. The interfacial tension can be determined if the drop length is at least four times longer than the drop radius: L > 4R (Equation (95)).

(95) $$\gamma =\frac{\Delta \rho \cdot R^{3}\cdot \omega^{2}}{4}$$

where **ω** is the angular velocity and **R** is the radius of the spinning drop.

To investigate the viscoelastic properties of systems with such low interfacial tension, the spinning drop is deformed with a known frequency instead of a steady rotational velocity, which directly results in a sinusoidal change of the interfacial area. In this instance, the interface elongation–compression is performed by oscillating the continuous phase. The calculation of the dilational modulus E_d_ and viscosity *η*_d_ is the same as explained previously.

The spinning drop method in oscillation was developed for the first time in 2018 by Zamora et al. [85], who used the oscillating spinning drop method to study surfactant-free systems, soluble surfactant systems and insoluble surface-active species (asphaltenes in a solvent–brine interface).

## 6. Conclusions and Perspectives

In this review, the theoretical and experimental aspects of interfacial rheology are highlighted. This particular branch of rheology has been used to study liquid–liquid or air–liquid systems with or without an active agent at the surface (or interface). In order to probe the interfacial viscoelastic properties and follow the same formalism as in bulk rheology, an interface must be deformed, either by shear force, in which the interfacial area remains constant, or by elongation, in which the interfacial area changes. Each type of deformation provides information about the surface (or interface), depending on the application studied.

As shown in the different parts of this report, interfacial shear rheology requires the use of a geometry positioned at the interface of the two sub-phases. The oscillating needle, the bicone and the double-wall ring (DWR) are the most common geometries used to probe the interface. Deforming an interface generates a flow at the sub-phase level, and the recorded response is the sum of the interfacial effects and the bulk effects; hence, the interest in quantifying a unitless macroscopic number, the Boussinesq number, which represents the ratio of these two effects. At low Boussinesq numbers, the subtraction of the contribution of the sub-phases is accomplished numerically with an iterative method (DWR and oscillating needle) or directly using an exact solution (bicone).

When the interface (or surface) has to be expanded (or compressed), interfacial dilatational rheology is involved. Several studies have been based on the Langmuir balance or the oscillating pendant drop method. The principle of these techniques is to measure the change in interfacial tension after a change in interfacial area, either via imaging (rising or pendant drop) or by measuring the surface pressure (Langmuir balance). The correction of the viscoelastic moduli by subtracting the effects of the sub-phase viscosity and elasticity has also been documented.

The oscillating drop method has potential for the study of the interfacial expansion of molten polymers if it can be adapted, in the same way as discussed in the case of the shear force, using a heated closed cell and isolation with suitable syringes and needles (low-capillary and Weber numbers).

The interfacial rheology of polymer systems in the molten state has rarely been investigated due to the difficulty of manipulating these materials and due to their intrinsic properties, such as their viscosity and high melting points. On the other hand, current commercial interfacial rheological devices have not been designed to study molten polymer systems, which require an adaptation of these techniques.

Probing the interfaces between two molten polymers requires the overcoming of technical or material constraints. A heating system is essential to broaden the range of polymers studied, as well as an innovative shear geometry that is both robust mechanically, to deform viscous polymer interfaces, and also light with a high geometric factor (Boussinesq number), to reduce its inertia when performing experiments within the limits of current rheometers and tensiometers.

Contrary to the conventional indirect modeling methods used for the assessment of polymer–polymer interfacial properties, the application of interfacial rheology to molten polymer systems might allow the direct measurement of their interfacial behavior, which is predominant in the case of polymer blends, coextruded multilayer polymers and polymer foams. Additionally, interfacial rheology can be essential to study interdiffusion and interphase formation from one polymer to another in the case of reactive interfaces, such as reactive compatibilized immiscible polymer melts. For all these reasons, high-temperature interfacial rheology appears to be one of the most promising techniques that should be further developed in the near future to probe the different polymer–polymer interfaces encountered in the processing and formation of multiphase polymer systems.

## Figures and Tables

**Table 1:** Geometrical ratios of interfacial shear geometries.

Geometry	DWR (TA Instruments)	Bicone (Anton Paar)	ISR (KSV)
$\frac{1}{l}\;\left(m^{-1}\right)$	1428.57	0.03	55.55

## Data Availability

No supporting data used in the study.
